# The Complex Association of FcγRIIb With Autoimmune Susceptibility

**DOI:** 10.3389/fimmu.2019.02061

**Published:** 2019-10-15

**Authors:** J. Sjef Verbeek, Sachiko Hirose, Hiroyuki Nishimura

**Affiliations:** Department of Biomedical Engineering, Toin University of Yokohama, Yokohama, Japan

**Keywords:** SLE, systemic lupus erythematosus, autoimmue disease, mouse model, Fcgamma receptor IIB, reverse genetics

## Abstract

FcγRIIb is the only inhibitory Fc receptor and controls many aspects of immune and inflammatory responses. The observation 19 years ago that *FcγRIIb*^−/−^ mice generated by gene targeting in 129 derived ES cells developed severe lupus like disease when backcrossed more than 7 generations into C57BL/6 background initiated extensive research on the functional understanding of this strong autoimmune phenotype. The genomic region in the distal part of Chr1 both in human and mice in which the *FcγR* gene cluster is located shows a high level of complexity in relation to the susceptibility to SLE. Specific haplotypes of closely linked genes including the *FcγRIIb* and *Slamf* genes are associated with increased susceptibility to SLE both in mice and human. Using forward and reverse genetic approaches including in human GWAS and in mice congenic strains, KO mice (germline and cell type specific, on different genetic background), knockin mice, overexpressing transgenic mice combined with immunological models such as adoptive transfer of B cells from Ig transgenic mice the involved genes and the causal mutations and their associated functional alterations were analyzed. In this review the results of this 19 years extensive research are discussed with a focus on (genetically modified) mouse models.

## Introduction

Antibodies (Ab) form immune complexes (IC) with their cognate antigen (Ag). IgG-ICs are potent activators of the immune system via cross-linking of receptors for the Fc part of IgG, FcγR, mainly expressed on the surface of cells of the innate immune system.

FcγRs belong to the Ig supergene family of leukocyte FcR and are transmembrane glycoproteins containing a ligand-binding α subunit with two or three extracellular Ig-like domains, a transmembrane and a cytoplasmic domain. In mice, the high-affinity FcγRI, binding monomeric IgG, and the low-affinity receptors for complexed IgG, FcγRIII, and FcγRIV are activating receptors. The α subunits of the activating receptors form a multi-subunit complex with a dimer of the common γ-chain (FcRγ) (1, 2) with an immunoreceptor tyrosine-based activation motif (ITAM). Cross-linking activating FcRs by IC initiates signal transduction via recruitment and subsequent activation of intracellular tyrosine kinases (3), switching on a large variety of effector mechanisms activating inflammatory cascades.

In humans, there are four activating FCGRs. The high-affinity FCGR1 (CD64) and the low-affinity FCGR3A (CD16A) are associated with the common γ chain whereas the low-affinity FCGR2A (CD32A), containing an ITAM in its cytoplasmic domain, and the low-affinity FCGR3B (CD16B), with a glycosylphosphatidylinositol (GPI) anchor, are single-chain receptors. All human FCGR genes are clustered at the distal end of Chr1, a region associated with susceptibility to autoimmune diseases such as Systemic Lupus Erythematosus (SLE) (4). In mice the FcγRII, -III, and -IV genes are clustered at the distal end of Chr1, a region orthologous with SLE associated genomic intervals on human Chr1 and associated also with susceptibility to autoimmune disease (Lupus-like disease). *FcγRI* is located on Chr3 due to a translocation during evolution after mouse and human had diverged.

In both humans and mice, the activating FcγRs are counterbalanced by one inhibitory single-chain low-affinity receptor FcγRIIb (FCGR2B or CD32B) with an inhibitory motif named immunoreceptor tyrosine-based inhibition motif (ITIM) within its cytoplasmic domain. In addition, co-engagement of FcγRIIb and the ITAM containing B-cell receptor (BCR) on B cells forms an important negative feedback mechanism to control antibody production. This regulatory mechanism of cellular activation by the ITAM-ITIM motif pair, observed originally with FcγR, has been described for many other receptors in the immune system e.g., T cell receptors and B cell receptors (5, 6). This review focuses on the important but still puzzling immune regulatory role of the inhibitory FcγRIIb and the complex association of its impaired function with autoimmunity as studied extensively in mice.

## General Characteristics of FcγRIIb

### Isoforms

In humans and mice, there are two membrane-bound isoforms of FcγRIIb identified: FcγRIIb1 and b2 (7) resulting from alternative splicing. The cytoplasmic domain is encoded by three exons whose 5′ exon encodes a 47 amino acid motif that prevents coated pit localization, which inhibits FcγRIIb mediated endocytosis of soluble immune complexes. This exon is present in the mRNA that encodes the b1 isoform, the only isoform expressed on B cells, but absent in the mRNA that encodes the b2 isoform (8, 9) expressed on most innate immune cells. The ITIM dependent inhibition of cell activation is the same for both isoforms. Therefore, the name FcγRIIb is used in this review without making a distinction between the b1 and the b2 isoform.

### Expression

In mice FcγRIIb is expressed on all innate immune cells and is the only FcγR expressed on B cells, including pre-, pro-, and mature B cells, memory B cells, plasma cells (10, 11) and B1 cells (12). Unlike many other B cell surface receptors, expression of FcgRIIb is not downregulated during plasma cell differentiation (10). FcγRIIb expression is modulated on different B cell subsets (11) and increases when the B cells become activated (11, 13). T cells do not intrinsically express FcγRs (14). However, it has been reported that expression of FcγRIIb but not any other FcγR, is upregulated in memory CD8^+^ T cells after *Listeria monocytogenes* infection and tempers the function of these cells *in vivo* (15). Guilliams et al. showed that according to the microarray expression values extracted from public data sets the mRNA expression of FcγRIIb in mice is from high to low as follows: Inflammatory macrophages (Mφ), Ly6C^hi^ classical monocyte, inflammatory monocyte-derived dendritic cell (moDC), lung CD11b^+^ conventional or classical DC (cDC), Ly6C^lo^ patrolling monocyte, alveolar Mφ, follicular B cell, GC B cell, skin-draining lymph node CD11b^+^ cDC, spleen CD8^+^XCR1^+^ cDC, spleen plasmacytoid DC (pDC), spleen CD11b^+^ cDC, neutrophils, spleen Mφ, and NK cells (16). The overall FcγRIIb expression pattern is similar in mouse and human. In mouse cDCs the relatively low expression of FcγRIIb is higher than that of any activating FcγR.

FcγRIIb expression, relative to that of activating FcγRs, is tightly regulated. In mice, C5a rapidly down-regulates FcγRIIb on alveolar Mφ and upregulates FcγRIII on these cells (17, 18). IL-4 downregulates FcγRIIb expression on mouse activated B cells (13, 19). IFNγ increases FcγRIIb expression on B cells (19) and increases the expression of activating FcγR on myeloid effector cells in mice. In humans the Th2 cytokines IL-4, IL-10, and TGF-β increase FCGR2B expression and decrease activating FCGR expression on myeloid cells (20–22) whereas IFNγ decreases FCGR2B expression on these cells and increases activating FCGR expression (23).

FcγRIIb is also expressed on non-hematopoietic cells. Its expression is induced on FDC upon antigen stimulation (24). It has been calculated that almost 70% of total mouse body FcγRIIb is expressed on liver sinusoidal endothelial cells (LSEC) (25, 26). On mouse glomerular mesangial cells, TNFα/IL-1β upregulates FcγRIIb expression whereas IFNγ downregulates FcγRIIb expression and upregulates the activating FcγR (27).

### Cellular Function

Co-aggregation of the inhibiting ITIM containing FcγRIIb with activating ITAM containing FcRs results in the recruitment of the inositol polyphosphate-5-phosphatase SHIP1 that counteracts the signals mediated by activating FcRs (3, 28). Therefore, FcγRIIb has a strong regulatory role in all the processes in which activating FcγR are involved. The ratio between activating and inhibiting signals determines the outcome of the cellular response to IgG-ICs. This ratio depends mainly on the following factors: (a) the relative affinities of the different antibody isotypes involved for the different FcγR, (b) the level of opsonization, and (c) the relative expression level of inhibitory and activating FcγR, which is partially determined by the cytokine milieu. The binding of FcγRIIb for IgG-IC is strongest for IgG1 and weakest for IgG2a. So, FcγRIIb expression has the highest impact on IgG1-IC. In addition, FcγRIIb can inhibit complement-mediated inflammation when co-engaged with Dectin-1 by galactosylated IgG1-ICs (29) indicating that its immune-modulatory function in the efferent response is not restricted to the regulation of activating FcγRs.

In B cells co-crosslinking of the BCR and FcγRIIb results in the inhibition of activation, proliferation, Ag internalization and Ab secretion (30–32). Moreover, *in vitro* studies have shown that FcγRIIb on B cells can induce apoptosis upon clustering (10, 12, 28, 33, 34).

FcγRIIb can also function as an endocytic receptor of small ICs. The endocytic properties of FcγRIIb depend on the presence of a di-leucine motif in the intracellular domain (8) and are independent of the ITIM.

### Role in Different Tissues and Cell Types

#### Myeloid Effector Cells

In the efferent phase, FcγRIIb sets a threshold for the activation by IgG-IC of myeloid effector cells, e.g., monocytes, Mφs, and neutrophils. Crosslinking of activating FcγR by IgG-ICs induces effector mechanisms of these cells e.g., soluble IC clearance, antibody-dependent cell-mediated cytotoxicity (ADCC), antibody-dependent cellular phagocytosis (ADCP), the release of inflammatory mediators, degranulation, superoxide production, enhancement of Ag presentation, and cell maturation and proliferation. This includes also the regulation of high-affinity IgE receptor-mediated mast cell activation (35).

Lupus-prone (NZBxNZW)F1 mice deficient for the FcR γ chain, lacking functional activating FcγR, do not develop IC-mediated severe glomerulonephritis (GN), despite high autoantibody titers (36). This suggests that FcγR play a dominant role in the efferent phase of Ab-driven diseases including lupus-like disease and therefore FcγRIIb might have a strong protective role in such a disease. In addition, FcγRIIb might also inhibit an ongoing auto-Ab response by suppressing the activating FcγR dependent, IgG-IC-triggered release of inflammatory mediators and other immune regulatory molecules by myeloid effector cells.

#### Dendritic Cells

DCs are central regulators of immunity determining whether tolerance is induced, or an effective adaptive immune response is generated, bridging innate and adaptive immunity (37–39). DCs have the unique capacity to take up exogenous Ag via a variety of mechanisms and surface molecules, including FcγR, and subsequently process and present the Ag-derived peptides in their MHC molecules to prime naïve T cells. Three main subsets of DCs can be recognized, cDC, moDC and pDC. Their ontogeny and functions have been reviewed extensively (40, 41).

A series of observations suggest that FcγR on cDCs and moDCs can play a role in priming and regulation of adaptive immunity (16). Ag-specific IgG enhances Ab responses to soluble protein Ag via activating FcγRs, probably by increasing Ag presentation by dendritic cells to Th cells (42). Many laboratories have shown that soluble IgG-ICs strongly enhance cross-presentation by using either *in vitro* assays (43–45), or *in vivo* assays with *in vitro* loaded DCs from WT and FcγR KO mice (46–50). Signaling through the activating FcγRs results in lysosomal targeting of the Ag and importantly activation and maturation of the DCs (44), required for their migration to the lymph node and their presentation of Ag-derived peptides in MHC class I to CD8^+^ T cells (49, 51). In mouse bone marrow-derived DCs (BMDCs), activating FcγRs modulate the expression of many genes, associated with T cell response induction, upon crosslinking by IgG-ICs. This is strongly regulated by FcγRIIb, setting a threshold for DC activation and maturation (52). *FcγRIIb*^−/−^ mice showed an increased upregulation of costimulatory molecules, resulting in an enhanced capacity to generate antigen-specific T cell responses upon injection of IgG-ICs (52–54). However, *in vivo*, in mice, the role of FcγR in the presentation of soluble IgG-IC derived Ag is redundant (55, 56). In mice, cDCs consist of two main subsets, type 1 cDC or cDC1 and Type 2 cDC or cDC2 (41). *In vivo* IgG-IC improve strongly cross-presentation of the cDC2 but not the cDC1 DCs. Only cDC2 mediated cross-presentation is FcγR dependent (57). Moreover, FcγRs are dispensable for the *in vivo* uptake of IgG-IC by cDC1 and cDC2 (56, 57). The *in vivo* cross-presentation of IgG-IC derived Ag by cDC1 is completely and by cDC2 partially dependent on C1q (56).

Because it has been shown that treatment with FCGR2B blocking antibodies results in spontaneous maturation of human DCs (58) it has been hypothesized that FCGR2B does not only regulate DC activation but also actively prevents unwanted spontaneous DC maturation by small amounts of circulating IC present in serum under non-inflammatory steady-state conditions (2).

IgG-ICs endocytosed by activating FcγR on DCs ends up in a degradative Lamp-1 positive compartment where it is slowly degraded into peptides (59). In contrast, antigen, endocytosed in the periphery via FcγRIIb on DCs, enters preferentially in a non-degradative Lamp-1 negative intracellular vesicular compartment, that recycles to the cell surface to transfer the native antigen via interaction with the BCR to B cells in the lymphoid organs. This indicates that DCs, migrating into extrafollicular areas (60) and the splenic marginal zones (MZ) (61), are not only important for the production of B cell activating components but also for the delivery of Ag to the BCR (62).

The question is whether in an autoimmune disease self-antigen containing IgG-IC can trigger DCs to promote autoreactive immune responses by presenting autoantigens or to release B and T cell activating cytokines and other stimulating factors breaking tolerance and whether FcγRIIb on DCs negatively regulates these processes. That is any way at a stage of the disease that some autoantibodies are already produced.

pDCs produce type I IFN in response to viral nucleic acids sensed through TLR7 and TLR9 (63, 64). Their main function is to control tolerance in the steady state (65, 66). Mouse pDCs express exclusively FcγRIIb (67). Conflicting results have been published regarding FcγRIIb facilitated T cell priming by mouse pDCs (56, 67, 68). *In vitro* uptake of IgG-ICs by mouse pDC is FcγRIIb dependent but does not promote Ag presentation to T cells (67), similarly to what has been shown with FcγRIIb mediated IC uptake in cDCs (62). In contrast, it has been reported that subcutaneous (s.c.) injection of *in vitro* IgG1-IC loaded pDCs induces strong Ag-specific CD4^+^ and CD8^+^ T cell responses although with lower efficiency than cDCs. The IgG1-IC-loaded pDC mainly promoted a Th2/tolerogenic environment *in vivo* (68). Human pDCs express besides low levels of FCGR2B, the activating FCGR2A and FCGR3B (16) and show FCGR2A dependent IgG enhanced Ag presentation to T cells (69). SLE patients have circulating ICs, containing small nuclear RNA and anti-small nuclear RNA IgG. pDCs can acquire such IC via FCGR mediated uptake resulting in stimulation of TLR7 and 8 and production of IFNα (70), a cytokine that is believed to play a central role in SLE pathogenesis (71). However, this requires FCGR2A and not FCGR2B (72). Therefore, it is unlikely that such a pathogenic process plays a role in lupus-like disease in mice.

#### B Cells and FDC

Primary B cells, developed and selected in the bone marrow, are recruited into GCs within the spleen and lymph nodes to undergo affinity maturation by Somatic Hypermutation (SHM). Three main mechanisms maintain self-tolerance in the primary B cell repertoire: central clonal deletion, receptor editing, clonal anergy induction (73). The first two effectively remove autoreactive B cells from the system. Clonal anergy occurs when self-reactive B cells interact with a self-Ag with relatively low avidity. The result is that BCR signaling is desensitized because of chronic exposure to self-antigens (74, 75) and differentiation into plasma cells is suppressed (76) resulting in the maintenance of anergic B cells with the potential to produce auto-Abs which can be recruited into GC (77). Anergic B cells can get T help if their BCR cross-reacts with foreign Ag but because of impaired BCR signaling FAS-mediated apoptosis is induced. However, extensive cross-linking by a foreign antigen can overcome the attenuated BCR signaling in anergic B cells inhibiting apoptosis (74). Autoreactive primary B cells can escape negative selection because of “clonal ignorance” when self-reactive B cells cannot encounter their self-Ag because it is hidden inside the cell. Development, responsiveness, and lifespan of ignorant cells is normal (76, 78, 79). The lack of T cell help after Ag contact induces apoptosis in ignorant self-reactive B cells in the periphery. However, it is striking that many auto-Abs are directed against intracellular Ags such as DNA. Therefore, it has been suggested that ignorant self-reactive B cells might be important for the development of SLE (77). So, the GC has to deal with three types of potential autoreactive B cells: anergic and ignorant, both recruited, and newly generated by somatic hypermutation in the GC reaction. Several mechanisms are in place in the GC to avoid the development of auto-Ab producing plasma cells. A very high concentration of self-Ag in the GC either overrules the binding of the BCR to foreign Ag presented by the FDC and apoptosis is induced, because of the lack of additional signals provided by the FDC (80), or/and blocks presentation of foreign Ag to follicular helper T cells (T_FH_), whose survival signals are required. Alternatively, self-reactive B cells can be maintained temporarily until their self-reactivity is abrogated by somatic hypermutation (SHM) (81). Ignorant self-reactive primary B cells, activated by cross-reactive foreign Ag, can enter the GC to get T_FH_ help (82) and subsequently, receptor editing by SHM can destroy self-recognition and improve specificity for foreign antigen. However, this appears not sufficient to prevent that autoreactive B cells escape negative selection in the GC and enter the AFC (antibody-forming cell) pathway. More downstream tolerance checkpoints are required.

In the GC Ag is presented to B cells on the cell surface of FDC, mainly in the form of CR1/2 bound C3d-coated ICs. FcγRIIb is expressed on both the GC B cell and the FDC. Although FcγRIIb is upregulated on FDC in GC compared to non-GC FDC, its expression is relatively low compared to CR2 expression. Therefore, it is unlikely that FcγRIIb plays a role in the capture and presentation of Ag early on in the GC response (83). It is unclear how a GC B cell becomes activated, because binding of its BCR to the Ag, within the FDC bound ICs, will also crosslink FcγRIIb on that B cell. It has been suggested that FcγRIIb expression on FDC competes with FcγRIIb expression on GC B cells by binding most of the Fc domains in the ICs (84). The outcome of co-engagement of BCR and FcγRIIb by ICs bound to FDC in GC might be dependent on the balance between concurrent activating and inhibiting signals, leading to stimulatory, inhibitory, or apoptotic responses (33, 85, 86). FcγRIIb might set a threshold for B-cell activation, that enables the selection of B cells with a BCR with sufficiently high affinity, to become activated. B cells with BCRs that have lost their affinity for the presented Ag during the process of affinity maturation by SHM will get only signals via crosslinking of FcγRIIb, which could result in induction of apoptosis as has been demonstrated *in vitro* (28, 33, 87, 88). In conclusion, the inhibitory FcγRIIb would be an important checkpoint for the deletion of potentially autoreactive B cells in the GC.

An additional apoptosis inducing mechanism in the bone marrow might also contribute to the control of autoreactive B cells (10). Long-lived plasma cells persist in the bone marrow. To provide room to newly generated plasma cells that migrate to the bone marrow after a new infection has occurred, a restricted number of plasma cells in the bone marrow has to be eliminated. Based on observations *in vitro* and *in vivo* in mice it has been hypothesized that plasma cells (which intrinsically lack BCR expression) are killed by apoptosis, induced by cross-linking of FcγRIIb highly expressed on these cells (10).

#### Non-immune Cells

On LSEC FcγRIIb might function as an endocytic scavenger receptor removing small IgG-IC from circulation to prevent systemic IC triggered inflammation (25). FcγRIIb on renal mesangial cells might protect against IgG-IC induced inflammation in the kidney (89). Both mechanisms might protect against the pathogenesis of IC-driven autoimmune diseases such as glomerulonephritis in SLE in the efferent phase. Because of the lack of an endothelial cell-specific Cre expressing strain that is not transcriptionally active during early hematopoiesis, required to generate endothelium-specific FcγRIIb deficient mice, the specific role of FcγRIIb on LSEC should be studied by applying transplantation of bone marrow from WT mice into lethally irradiated *FcγRIIb* KO mice.

## Forward Genetics: Association of Autoimmunity and FcγRIIb Polymorphism

### In Mice

**Figure 1:**
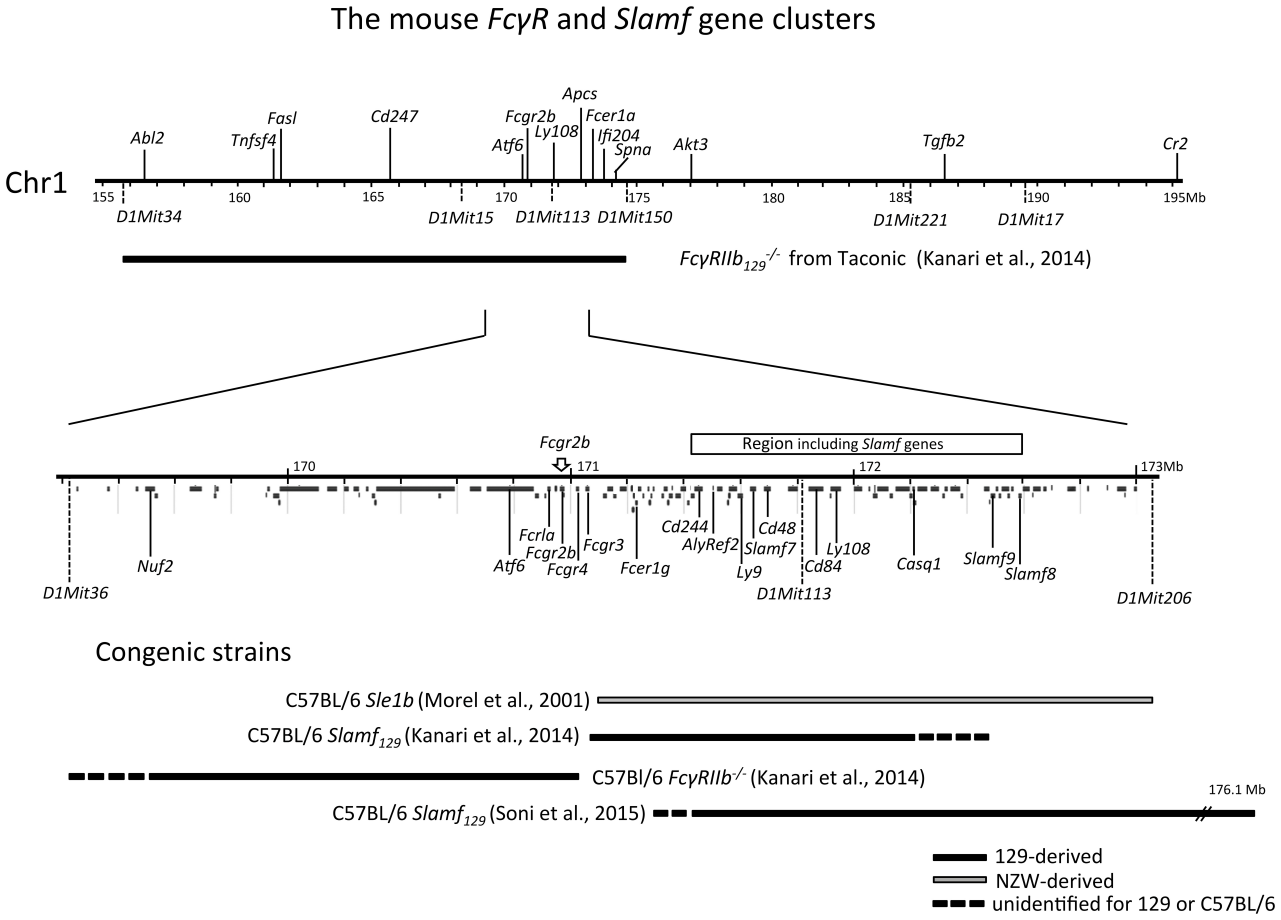
Physical map of the sub-telomeric region of mouse chromosome 1. The upper horizontal line represents a 40 Mb genomic region including the 129 derived *FcγRIIb* flanking region present in the original *F*γ${RIIb}_{129}^{-/-}$ mouse, backcrossed more than 7 generations into C57BL/6 background. This *FcγRIIb* flanking region spans at a minimum the distance between the microsatellite markers *D1Mit34* and *D1Mit150* [horizontal black bar; (98)]. The lower horizontal line represents a magnification of the 3.8 Mb subregion located between microsatellite markers *D1Mit36* and *D1Mit206* showing a detailed map of the *FcγR* and *Slam* family gene clusters within this region. In addition, below the line, the location of all other coding genes in this region is shown according to the NCBI database. At the bottom, the congenic fragments present in the different *FcγRIIb*^−/−^ and *Slamf*_129_ C57BL/6 congenic strains, described in the text, are depicted as horizontal bars.

The association between autoimmunity and *FcγRIIb* polymorphism is extensively studied in NZW and NZB inbred stains. NZB mice show limited autoimmunity (90) while NZW mice are not autoimmune although their B cells have intrinsic defects sufficient to break tolerance to nuclear antigens (91, 92). However, the (NZBxNZW)F1 offspring of an accidental cross between NZW and NZB mice (93) showed a severe lupus-like phenotype characterized by a gender-bias, expansion of activated B and CD4^+^ T cells, splenomegaly, elevated serum ANA and IC-mediated GN causing renal failure and premature death at 10–12 months of age (94). By backcrossing (NZBxNZW)F1xNZW followed by brother-sister mating the NZM2410 recombinant inbred strain with a homozygous genome was generated (95–97). In this mouse four SLE susceptibility loci, *Sle1-4*, have been identified on different chromosomes. *Sle1* is located on the telomeric region of Chr1 syntenic to human 1q23 that has shown strong linkage to SLE susceptibility in all human studies. The *FcγR* gene cluster maps in this region (Figure 1) and is from NZW origin in NZM2410 mice. From the NZM2410 strain, C57BL/6 strains have been developed congenic for a single SLE susceptibility locus. The presence of *Sle1* appeared to be sufficient to break tolerance in C57BL/6 mice and to drive the production of high titers of anti-chromatin ANAs with a selective Ab reactivity to H2A/H2B/DNA sub-nucleosomes (99, 100). Importantly, this step appears to be necessary for the induction of disease (100) making *Sle1* a key locus in the initiation of SLE. Transplantation of hematopoietic stem cells from C57BL/6 *Sle1* congenic mice into C57BL/6 recipient mice showed that *Sle1* causes independent B and T cell-intrinsic effects on the B cell response (101, 102).

Three *FcγRIIb* haplotypes [numbered I-III according to Jiang et al. (103), Table 1] have been recognized in inbred strains of mice and wild mice with variation in the promoter region and intron 3 (Table 1). Haplotype I with 2 deletions in the promoter region and one in intron 3 is found in autoimmune-prone strains and most wild mice and is associated with decreased expression of FcγRIIb on Mφ, activated B cells and GC B cells (11, 103–105). By using C7BL/6 congenic strains with the NZW (106) and NZB (107) allelic variants of *FcγRIIb* the effect of the deletions in haplotype I and II on B cell expression was studied. When immunized with KLH, FcγRIIb expression on splenic non-GC B cells was high and similar in C7BL/6 and C57BL/6 *FcγRIIb*_*NZB*_ congenic mice. In contrast, the expression on activated GC B cells was markedly down-regulated in C57BL/6 congenic *FcγRIIb*_*NZB*_ mice and up-regulated in control C57BL/6 mice, in comparison with the expression levels on non-GC B cells. The downregulation of FcγRIIb expression on activated GC B cells was associated with an increase of IgG anti-KLH Ab titers. C57BL/6 *FcgRIIb*_*NZB*_ congenic mice also showed lower FcγRIIb expression on Mφ compared with WT C57BL/6 mice (107). In a C57BL/6 knockin (KI) mouse model of the 5' region of the haplotype I *FcγRIIb* gene (*FcγRIIb*_*NZB*_), FcγRIIb failed to be upregulated on activated and GC B cells resulting in enhanced early GC responses and low auto-Ab production without kidney disease as discussed later in more detail (11).

**Table 1:** Allelic variants of mouse *FcγRIIb* gene and their association with impaired expression and autoimmune disease susceptibility.

**Haplotype**	**Mouse strain**	**Genetic variation**	**Phenotype**
I	NZB, BXSB, MRL, NOD, Wild mice 129	13 bp 5′ deletion in promoter 3 bp 3′ deletion in promoter 4 bp 5′ deletion in intron 3	Decreased expression on Mφ and activated and GC B cells. Autoimmune-prone (except 129)
II	NZW, SWR, SJL	4 bp 5′ deletion in intron 3 24 b 3′ deletion in intron 3	Decreased expression on GC B cells. Potential to accelerate autoimmunity
III	C57BL/6, BALB/c, DBA	No deletions	Not autoimmune

As mentioned earlier, *in vitro* cross-linking of FcγRIIb on B cells from C57BL/6 mice can induce apoptosis. However, plasma cells from autoimmune-prone NZB or MRL mice could not be killed *in vitro* by FcγRIIb cross-linking because of too little expression of the receptor (10). This might partially explain why these autoimmune-prone mice have larger numbers of plasma cells and might contribute to the autoimmune phenotype of these mice.

**Figure 2:**
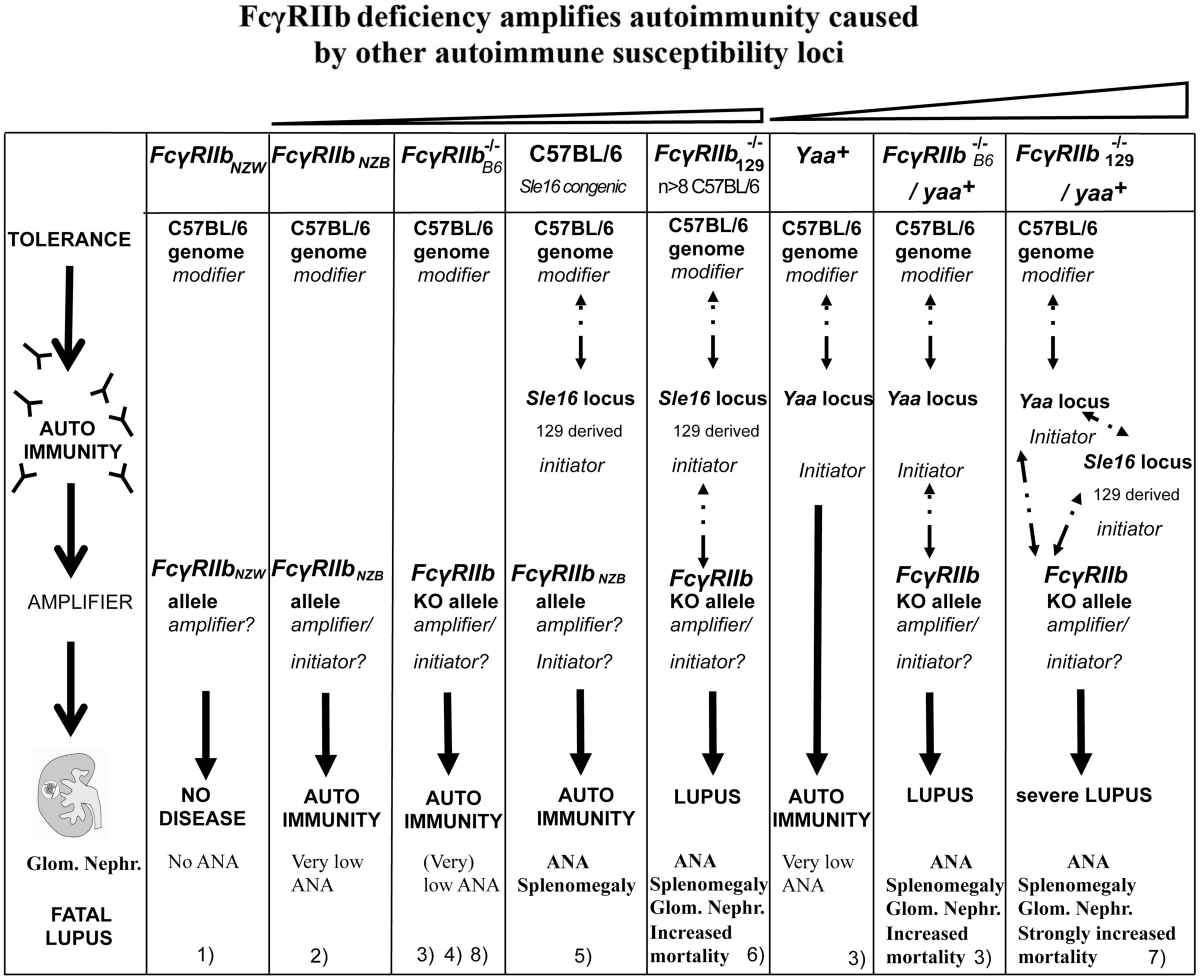
Epistasis between the *FcγRIIb* KO alleles and the *Sle16 (Slam*_129_) and *Yaa* autoimmune susceptibility loci resulting in lupus-like disease in C57BL/6 mice. Epistatic interactions are indicated as dotted arrows. The FcγRIIb flanking *Sle16* genomic region contains the autoimmunity associated *Slamf*_129_ haplotype 2 gene cluster (see Figure 1). (1) Rahman et al. (108); (2) Espéli et al. (11); (3) Boross et al. (109); (4) Li et al. (110); (5) Bygrave et al. (111); (6) Bolland and Ravetch (112); (7) Bolland et al. (113); (8) Kanari et al. (98). The increasing severity of autoimmune disease in the different mouse models is depicted on top.

Similarly, to the *FcγRIIb*_*NZB*_ allele, the *FcγRIIb*_*NZW*_ allele in the C57BL/6 *Sle1* congenic strain did not upregulate its expression on GC B cells and plasma cells, as did the C57BL/6 allele, when immunized with SRBCs. However, in the absence of its *Sle1* flanking regions, *FcγRIIb*_*NZW*_ did not induce an autoimmune phenotype but was associated with an increased number of class-switched plasma cells (108). This might indicate that the decreased expression of the *FcγRIIb*_*NZW*_ allele is not sufficient for the development of autoreactive B cells but can result in the increase of the number of autoreactive B cells, induced by other lupus-susceptibility loci, by enhancing the production of class-switched plasma cells. This suggests that the *FcγRIIb*_*NZB*_ (haplotype I) allele has a stronger impact on susceptibility to autoimmunity than the *FcγRIIb*_*NZW*_ (haplotype II) allele (Figure 2). However, in one study comparing the phenotypes of C57BL/6 strains congenic for different intervals of the *Nba2* locus, a region on Chr1 of NZB mice corresponding to the *Sle1*_*NZW*_ locus, *FcγRIIb*_*NZB*_ was identified as an autoimmune susceptibility gene (114), in another it was not (115). *Sle1* can be divided in four non-overlapping sub-loci: *Sle1a, -b, -c*, and *-d. Sle1b* is far the most potent autoimmune susceptibility locus causing almost the same phenotype as the whole *Sle1* locus: gender-biased spontaneous loss of immune tolerance to chromatin, the production of high titers of IgG auto-Abs with a penetrance of 90% at 9 months of age and increase of total IgM and B7-2 expression on B cells (116). This suggests that *Sle1b* mainly affects B cells. The genomic location of *Sle1b* was determined by phenotypic analysis (e.g., ANA production) of a series of C57BL/6 congenic strains carrying truncated *Sle1* intervals. C57BL/6 congenic mice with an NZW derived genomic fragment, containing the *FcγR* cluster, did not develop ANA whereas C57BL/6 mice, containing an adjacent 900 kb congenic NZW fragment expressing 24 genes including seven members of the highly polymorphic signaling lymphocytic activation molecules (*Slam*) cluster, did. This positions the *FcγR* cluster just outside the *Sle1b* locus (117) and confirms previous observations that *FcγRIIb* is located in between the *Sle1a* and *Sle1b* loci (113) (Figure 1). Together these data suggest that in C57BL/6 *Sle1* congenic mice the *FcγRIIb*_*NZW*_ allele is not required for the development of an autoimmune phenotype, whereas the adjacent *Slam* cluster is. Because of these puzzling results, the questions remain why FcγRIIb is upregulated on GC B cells in non-autoimmune inbred strains such as C57BL/6 and BALB/c and why this is impaired in autoimmune-prone mouse strains and how does that contribute to the autoimmune phenotype of these mice.

*Slam* family (*Slamf*) member genes encode cell surface glycoproteins with extracellular binding domains that mediate stimulatory and/or inhibitory signaling via associations with members of the Slam-associated protein (SAP) family of signaling adaptors during cell-cell interactions between many hematopoietic cell types (118–120). They are the only genes within the *Sle1b* interval with obvious immunological functions (117). Most Slamf members act as self-ligand and are expressed on many lymphoid and myeloid cell subsets, platelets, and hematopoietic stem and progenitor cells. Slamf plays a role in the interaction of CD4^+^ T cells with cognate B cells, recruitment and retention of T cells within the emerging GCs (121–123), long-lasting T cell:B-cell contact, optimal T_FH_ function, T cell activation (124, 125), stabilization of B–T cell conjugates and sustaining effective delivery of T cell help required for GC formation (126, 127).

The *Slamf* genes show extensive polymorphisms (117) but only two haplotypes of the *Slamf* locus have been identified in laboratory mouse strains. Haplotype 1 is represented by C57BL/6 and related strains and haplotype 2 by all autoimmune-prone mouse strains, as well as many non-autoimmune mouse strains including BALB/c and 129. The polymorphism in *Slamf* member *Ly108* affects the expression of two alternatively spliced isoforms, Ly108-1 and, Ly108-2, which differ exclusively in their cytoplasmic region (117). Ly108-1 is dominantly expressed in T and B lymphocytes of mice with haplotype 2, whereas Ly108-2 is dominantly expressed in T and B cells of mice with haplotype 1. Modulation of the BCR signaling by Ly108-1 results in the impaired negative selection of B cells (128). Overexpression of both C57BL/6 derived non-autoimmune Ly108 and CD84 Slamf members was required to restore tolerance in autoimmune-prone C57BL/6 *Sle1* congenic mice (129), indicating that polymorphism in both *Slamf* genes contributes to the autoimmune phenotype of C57BL/6 *Sle1* congenic mice.

In the NZM2410 model four NZW-derived SLE suppressor loci have been identified (130). The presence of such suppressor loci might explain why NZW and also129 and BALB/c mice do not develop autoimmune disease, although they carry the type 2 *Slamf* haplotype.

### In Humans

The reported copy number variation (CNV) in human *FCGR* genes does not involve *FCGR2B* (131–134). A series of single nucleotide polymorphisms (SNPs) have been reported to be located both in the promoter and the encoding region of the human *FCGR2B* gene (135). Two SNPs are located in the promoter region at nucleotide positions−386 and −120 (−386G>C; *rs3219018* and −120A>T; *rs34701572*) (136) resulting in four haplotypes:−386G−120T (named *FCGR2B.1*), −386C−120T (*FCGR2B.2*),−386G−120A (*FCGR2B.3*), and −386C−120A (*FCGR2B.4*). The rare *FCGR2B.4* haplotype increased the transcription of *FCGR2B in vitro* and resulted in increased FCGR2B expression on EBV transformed B cells and primary B cells (137) and myeloid cells (138), compared to the more frequent *FCGR2B.1* haplotype. However, independently, others have shown that homozygosity of the −386C genotype decreases the transcription and surface expression of FCGR2B in peripheral B cells compared to the −386G homozygote genotype (139). Up till now, there is no explanation for these contradictory results.

In the transmembrane encoding fifth exon a non-synonymous C to T transition was identified, *rs1050501*, resulting in the substitution of isoleucine with threonine at position 232 (140), excluding the receptor from lipid rafts. This prevents interaction of FCGR2B with ITAM containing receptors such as the activating FCGR and the BCR (141, 142). Mφs from individuals homozygous for *FCGR2B*^*T*232^ showed a stronger phagocytic capacity of IgG-IC while the B cells of these individuals showed reduced FCGR2B-mediated inhibition of BCR-triggered proliferation (142).

GWAS analyses have shown an association between *rs1050501* and SLE (140, 143–147). Three meta-analyses confirmed these associations (147–149). The *FCGR2B*^*T*232^ homozygosity is associated with an odds ratio of 1.73, one of the strongest associations in SLE (147). Association of *rs1050501* with Rheumatoid Arthritis (RA) has been reported for a Taiwanese cohort (150).

The frequency of homozygosity of the *FCGR2B*^*T*232^ allele is only 1% in Caucasians and in contrast 5–11% in African and South-East Asian populations (151). This might be one of the explanations for the ethnic differences in SLE susceptibility. Malaria is endemic in Africa and South-East Asia. An association was found between decreased susceptibility for severe malaria and homozygosity for the *FCGR2B*^*T*232^ allele (135). So, increased protection against malaria by down-regulation of FCGR2B expression goes along with increased risk to develop SLE.

A significant but weak association has been observed between *SLAMF* and susceptibility to SLE. The weakness of the association might be explained by the limited size of the cohorts studied (152). An association study of UK and Canadian families with SLE has revealed multiple polymorphisms in several *SLAMF* genes (153). However, the strongest association with a non-synonymous SNP could not be replicated in independent Japanese and European cohorts of SLE patients (154, 155). Instead, another SNP was significantly associated with the susceptibility to SLE in another Japanese cohort (156). One large-scale case-control association study showed an association of two SNPs with increased susceptibility to RA, in two independent Japanese cohorts (155). In conclusion, these observations indicate that also in human's polymorphisms of *SLAMF* contribute to the susceptibility to autoimmune disease.

Overall, a model emerges from both studies with C57BL/6 *Sle* congenic mouse strains and human SLE (157), in which disease susceptibility arises through the co-expression of multiple genetic variants that have weak individual effects (152, 158). According to the “threshold liability” model, the severity of the autoimmune phenotype increases with the increasing number of autoimmunity associated allelic variants of autoimmune susceptibility genes in the genome. However, epistatic interactions might result in a more complex non-additive inheritance of the autoimmune phenotype (Figure 2). According to this “multiplicative model” the interactions of all susceptibility and suppressor alleles in the genome determine the susceptibility for autoimmune diseases of an individual (159). Importantly this means that the contribution of an individual gene to the autoimmune phenotype can vary depending on the presence of other susceptibility and suppressor genes in the genome (the genomic context). This might explain the puzzling and contradictory results with the *FcγRIIb*_*NZW*_ and *FcγRIIB*_*NZB*_ haplotypes. To uncover the polygenic effects associated with a complex disease such as SLE not a single gene association approach but gene set analysis (GSA) is required (160). However, a reverse genetic approach might offer the opportunity to reconstruct an autoimmune phenotype by modifying a combination of a limited number of candidate genes in a well-defined genetic background.

## Reverse Genetics

So far three *FcγRIIb* KO mouse models have been published. The first published KO was generated by gene targeting in 129 derived ES cells (161) and subsequently backcrossed into the C57BL/6 background, here called *Fcγ*${RIIb}_{129}^{-/-}$ mouse. This mouse on a not well-defined mixed genetic background was during 15 years (between 1996 and 2011) the only *FcγRIIb* KO model available and has been extensively used resulting in an overwhelming amount of literature concerning the role of FcγRIIb in immune tolerance. Subsequently, independently, in two different laboratories *FcγRIIb* KO mice were generated by gene targeting in C57BL/6 derived ES cells, here called *Fcγ*${RIIb}_{B6}^{-/-}$ mice (109, 110). The published data regarding ANA titers of one of these mouse strains are inconsistent (162, 163) as are the autoimmune phenotypes of both C57BL/6 strains (109, 110). Moreover, it is still under debate to what extent the autoimmune phenotypes of the *Fcγ*${RIIb}_{B6}^{-/-}$ mice differ from the autoimmune phenotype of the *Fcγ*${RIIb}_{129}^{-/-}$ mice. Therefore, we discuss in chronological order these different models.

### The *FcγRIIb* KO Mouse on Mixed 129/C57BL/6 Background

The *Fcγ*${RIIb}_{129}^{-/-}$ mouse develops elevated immunoglobulin levels in response to both T cell-dependent and T cell-independent Ags (161), have more plasma cells (10), and show an enhanced passive cutaneous anaphylaxis compared to WT controls (161). They develop arthritis (164) and Good pasture's syndrome-like disease (165) upon immunization with bovine collagen type II and type IV, respectively when backcrossed into the non-permissive (*H-2*^*b*^ haplotype) C57BL/6 background. When backcrossed more than 7 generations into C57BL/6, but not BALB/c background, the *Fcγ*${RIIb}_{129}^{-/-}$ mice started to develop spontaneously with high penetrance lupus-like disease. This autoimmune disease is characterized by gender bias, splenomegaly, increase of the proportion of different subsets of activated lymphocytes with age, high titers of ANA, IC-mediated GN and vasculitis in different organs resulting in proteinuria and premature death (112) very similar to the phenotype of the NZM2410 mouse we discussed earlier. This is surprising because, as we have seen, genetic studies revealed that lupus susceptibility is a multigenic phenotype. Monogenic autoimmune diseases are rare (158). However, the strong autoimmune phenotype of the *Fcγ*${RIIb}_{129}^{-/-}$ mouse cannot be attributed exclusively to the deletion of the *FcgRIIb* alleles. This mouse has been generated by gene targeting in 129 derived ES cells and subsequently backcrossed into C57BL/6 background. Such a mouse is, even after 10–12 generations, not fully C57BL/6 but congenic for the 129 derived flanking regions of the targeted allele, containing still hundreds of genes of 129 origin (Figure 1). The 129 genome contains more than 1,000 non-synonymous mutations compared to the C57BL/6 genome (166). This is only one part of the problem. Epistasis between 129 derived loci and the C57BL/6 genome also occurs. It has been shown that mice without targeted alleles but congenic for the 129 derived distal-region of Chr1 (*Sle16*), a lupus-associated region including the autoimmune-prone haplotype 2 of the *Slamf* genes and the haplotype I of the *FcγRIIb* gene, develop a similar autoimmune phenotype as C57BL/6 *Sle1* congenic mice (111). That might explain why several mouse strains generated by targeting genes in the proximity of the *Slamf* locus in 129 derived ES cells, and backcrossed into C57BL/6 background, develop autoimmunity.

Strikingly, the *Fcγ*${RIIb}_{129}^{-/-}$ mouse backcrossed more than seven generations into C57BL/6 background develops ANA with similar selective reactivity to H2A/H2B/DNA sub-nucleosomes as C57BL/6 *Sle1* congenic mice, however, with earlier onset, stronger penetrance, and higher titers. Irradiated *Rag*^−/−^ C57BL/6 or *IgH*^−/−^ C57BL/6 mice adoptively transferred with bone marrow from *Fcγ*${RIIb}_{129}^{-/-}$ mice backcrossed more than seven generations into C57BL/6 background developed anti-chromatin antibodies and proteinuria, indicating that the disease is fully transferable, dependent on B cells. Myeloid *FcγRIIb* ^−/−^ cells are not required (112). This is in keeping with experiments, mentioned earlier, that show that the autoimmune phenotype of C57BL/6 *Sle1* congenic mice is completely reconstituted in C57BL/6 irradiated mice that received bone marrow from C57BL/6 *Sle1* congenic mice but not by the reciprocal reconstitution. This demonstrates that *Sle1* is functionally expressed in B cells (101) although impaired FcγRIIb expression seems to play a minor role in that model (113, 117). Taken together these data all point in the same direction: the strong lupus-like phenotype of the *Fcγ*${RIIb}_{129}^{-/-}$ mice backcrossed more than seven generations into C57BL/6 background is caused by epistatic interaction between the *Slamf*_129_ locus, the C57BL/6 genome, and *FcγRIIb*^−/−^ (Figure 2), similar to the epistatic interactions between *FcγRIIb*_*NZB*_ (haplotype I), *Slamf*_*NZB*_ (haplotype 2) and the C57BL/6 genome in C57BL/6 *Nba2* congenic mice (114). As a consequence, the *Fcγ*${RIIb}_{129}^{-/-}$ mouse suffers from the confounding effect that the *FcγRIIb*_129_KO alleles are closely linked to the *Slamf*_129_ locus associated with autoimmunity. This means that in most experimental conditions, no distinction can be made between *FcγRIIb*^−/−^ and *Slamf*_129_ mediated effects in these mice.

*Ig* gene analysis of ANA suggests that ANA develop in GCs (167–172). Therefore, analysis of the loss of tolerance in *Fcγ*${RIIb}_{129}^{-/-}$ mice focused on GC (173). The role of FcγRIIb as an immune tolerance checkpoint has been studied in a transgenic mouse model in which the variable heavy chain (V_H_) *3H9H-56R*, derived from a dsDNA specific hybridoma, or its variant *56RV*_*H*_, with higher affinity binding to dsDNA, were inserted in the *Igh* locus (*IgM*^*a*^ allele) (174). Receptor editing, based on the use of specific light chains that abrogates the dsDNA binding, is the main mechanism to maintain tolerance in these mice (175–177). The Ab selection process was compared between WT C57BL/6 and *Fcγ*${RIIb}_{129}^{-/-}$ mice carrying the *V*_*H*_ transgenes (178). C57BL/6 mice expressing the high-affinity *56R* allele (B6*.56R*) developed low but significant anti-DNA titers, indicating that tolerance was broken, whereas C57BL/6 mice with the low-affinity *3H9* allele (B6*.3H9*) did not. Tolerance was also maintained in *Fcγ*${RIIb}_{129}^{-/-}$ mice carrying the low-affinity *3H9* allele (*Fcγ*${RIIb}_{129}^{-/-}$*.3H9*). The development of IgM-positive autoreactive B cells was similar in *Fcγ*${RIIb}_{129}^{-/-}$ mice carrying the high-affinity *56R* allele (*Fcγ*${RIIb}_{129}^{-/-}$*.56R*) and B6.*56R* mice. Moreover, *Fcγ*${RIIb}_{129}^{-/-}$*.3H9* mice and *Fcγ*${RIIb}_{129}^{-/-}$*.56R* mice did not show differences in the populations of activated and GC B cells or T cells compared to B6.*3H9* and B6.*56R* control mice. However, *Fcγ*${RIIb}_{129}^{-/-}$*.56R* mice developed higher IgG anti-DNA titers compared to B6.*56R* mice. Taking together these observations suggest that the function of FcγRIIb in B6.*56R* mice is limiting the production of serum IgG anti-dsDNA. Analysis of hybridomas derived from these different mouse strains showed that a much higher percentage of hybridomas from *Fcγ*${RIIb}_{129}^{-/-}$*.56R* mice secreted IgG antibodies compared to the hybridomas from B6.*56R* mice. Moreover, *Fcγ*${RIIb}_{129}^{-/-}$*.56R* mice had a higher percentage of splenocytes with a plasma cell phenotype compared to B6.*56R* mice. The cross of the *Fcγ*${RIIb}_{129}^{-/-}$ mice with autoimmune B cell receptor transgenic mice most likely bypasses the involvement of Slamf_129_ (which is mainly responsible for the spontaneous development of autoreactive B cells in a C57BL/6 *Slamf*_129_ congenic strain, as we will see later). So, in this case, the phenotype of the *Fcγ*${RIIb}_{129}^{-/-}$*. 56R* mouse can be completely attributed to the absence of FcγRIIb. From these results, it was concluded that the main function of FcγRIIb in the GC reaction is to control, as one of the latest checkpoints, the development of autoreactive IgG-secreting plasma cells and that most likely FcγRIIb deficiency modifies autoimmunity rather than initiates loss of tolerance (178). This was confirmed independently, in an experimental model with two V_H_ chain knockin strains, HKI65 and HKIR, with specificity for the hapten arsonate and a weak and strong specificity for DNA respectively (179). No indications for a role of FcγRIIb in primary or GC tolerance checkpoints were found. Only an increased number of plasma cells was detected in mice that received C57BL/6 *HKIR/Fc*γ${RIIb}_{129}^{-/-}$ B cells. FcγRIIb seems to prevent autoimmunity by suppressing the production of autoreactive IgG from B cells that escaped negative selection in GC and enter the AFC pathway (179). This is also in agreement with observations in C57BL/6 *FcγRIIB*_*NZW*_ congenic mice mentioned earlier (108). However, more recently it has been shown that the number of spontaneous (Spt) GC B cells is increased in 6–7 months old *FcγRIIb*^−/−^ mice on a pure C57BL/6 background, suggesting that FcγRIIb deficiency dysregulates the Spt-GC B cell response [(163); **Table 3**] as will be discussed later.

The view that FcγRIIb acts as a suppressor of autoimmunity caused by other loci is supported by the observed synergism between *FcγRIIb*^−/−^ and several autoimmune susceptibility loci. Just like the *Sle1* locus (100), *FcγRIIb*^−/−^ interacts synergistically with the autoimmune susceptibility *Yaa* locus from BXSB autoimmune-prone mice, containing the *Tlr7* gene translocated from the X chromosome to the Y chromosome, resulting in strong acceleration of lupus-like disease in *Yaa*^+^*Fcγ*${RIIb}_{129}^{-/-}$ male mice (113) (Figure 2). MRL/*Fas*^*lpr*/*lpr*^ mice develop lupus-like disease whereas C57BL/6 *Fas*^*lpr*/*lpr*^ mice do not, likely due to suppressor activity of the C57BL/6 genome. However, C57BL/6 *Fas*^*lpr*/*lpr*^*Fcγ*${RIIb}_{129}^{-/-}$ mice develop systemic autoimmune disease (180). This is consistent with the presence of the haplotype I allelic variant of *FcγRIIb* in MRL mice with an impaired expression on B cell subsets. Mice deficient for both, deoxyribonuclease 1 like 3 (DNASE1L3) and FcγRIIb exhibit at the age of 10 weeks an IgG anti-dsDNA production higher than in 9 months old (NZBxNZW)F1 mice (181). The presence of either the *Yaa* locus or homozygosity for the *Fas*^*lpr*^ or *Dnase1l3* KO alleles is most likely sufficient to break tolerance. However, FcγRIIb prevents strong autoimmunity by suppressing the production of autoreactive IgG from B cells that have escaped negative selection and enter the AFC pathway. Because *Fcγ*${RIIb}_{129}^{-/-}$ mice were used in the crosses mentioned a role for Slamf_129_ cannot be excluded in these models as indicated by the much milder phenotype of the *Yaa*^+^ *Fcγ*${RIIb}_{B6}^{-/-}$ mouse on pure C57BL/6 background discussed later (109) compared to the severe lupus phenotype of the *Yaa*^+^*Fcγ*${RIIb}_{129}^{-/-}$ mouse (Figure 2). Nevertheless, these observations underscore the crucial role of FcγRIIb in the protection against the development of spontaneous autoimmunity determined by other autoimmune susceptibility loci.

Because of allelic exclusion, Ig transgenic mice do not have a normal B cell repertoire. Therefore, the development of self-reactive GC B cells and plasma cells was studied in *Fcγ*${RIIb}_{129}^{-/-}$ mice by large scale Ig cloning from single isolated B cells to determine how loss of FcγRIIb influences the frequency at which autoreactive ANA-expressing B cells participate in GC reactions and develop in plasma cells under physiological conditions (173). In comparison with WT controls the following was observed in *Fcγ*${RIIb}_{129}^{-/-}$ mice: (a) No skewing of *Ig* gene repertoire but enrichment for IgGs with highly positively charged IgH CDR3s which is associated with antibody autoreactivity; (b) lower numbers of somatic mutation; (c) increased numbers of polyreactive IgG^+^ GC B cells and bone marrow plasma cells and (d) enrichment of nucleosome-reactive GC B cells and plasma cells. The overall frequency of ANAs was high in GC B cells but not in plasma cells. These results demonstrate that in *Fcγ*${RIIb}_{129}^{-/-}$ mice IgG autoantibodies including ANAs are expressed by GC B cells and that somatic mutations contribute to the generation of high-affinity IgG antibodies suggesting that the *FcγRIIb*^−/−^*/Slamf*_129_ combination plays an important role in the regulation of autoreactive IgG^+^ B cells which develop from non-self-reactive or low-self-reactive precursors by affinity maturation (173). It would be of great interest to repeat this analysis in *Fcγ*${RIIb}_{B6}^{-/-}$ mice on pure C57BL6 background and C57BL/6 *Slamf*_129_ congenic mice to define the individual contribution of the *Slamf*_129_ locus and the *FcγRIIb* KO alleles in the loss of immune tolerance in the C57BL/6 background. Interestingly the frequency of high-affinity autoreactive IgG^+^ plasma cells was relatively low, given the high frequency of autoreactive IgG^+^ GC B cells. This can be explained by the existence of a tolerance checkpoint before GC B cells differentiate into spleen or bone marrow plasma cells, downstream of FcγRIIb and Slamf (173).

Complementation of the mutant phenotype of an organism by expression of a transduced WT gene is considered as the ultimate proof that the mutated gene is the cause of the phenotype. Irradiated autoimmune-prone BXSB, NZM2410, and *Fcγ*${RIIb}_{129}^{-/-}$ mice transplanted with autologous bone marrow transduced with a viral vector expressing FcγRIIb showed reduced autoantibody levels and as a consequence much milder disease symptoms compared to mice that received autologous bone marrow transduced with an empty vector (182). These results were confirmed by using a transgenic mouse with a stable 2-fold B cell-specific overexpression of FcγRIIb (183). These mice hardly developed a lupus-like disease when backcrossed into autoimmune-prone MRL/*Fas*^*lpr*/*lpr*^ background. The underlying mechanism of these strong effects of overexpression of FcγRIIb is not known. These experiments mainly demonstrate that overexpression of FcγRIIb on B cells inactivates these cells resulting in a strong decrease in autoantibody production. Although they confirm a role of FcγRIIb in autoimmune disease they don't answer the intriguing question whether FcγRIIb deficiency is a modifier of autoimmunity rather than a primary initiator of the loss of tolerance.

### *FcγRIIb* KO on a Pure C57BL/6 Background

To avoid the confounding effect of 129 derived flanking sequences (*Sle16*), independently, in two different laboratories *FcγRIIb*^−/−^ mice were generated by gene targeting in C57BL/6 ES cells. To distinguish between these two models, one is called here ^*Le*^*Fcγ*${RIIb}_{B6}^{-/-}$ (109) and the other ^*NY*^*Fcγ*${RIIb}_{B6}^{-/-}$ (110). ^*Le*^*Fcγ*${RIIb}_{B6}^{-/-}$ mice exhibit a hyperactive phenotype in the effector phase, although somewhat milder than *Fcγ*${RIIb}_{129}^{-/-}$ mice, suggesting a contribution of *Sle16* to the phenotype of the *Fcγ*${RIIb}_{129}^{-/-}$ mouse in the effector phase (109). Both KO mice develop very mild lupus-like disease (Table 2). Total IgG ANA was not significantly increased in 10 months old female ^*Le*^*Fcγ*${RIIb}_{B6}^{-/-}$ mice compared to C57BL/6 mice although serum of 5% of these mice showed some total IgG anti-dsDNA and anti-ssDNA antibody titers just above (C57BL/6) baseline. In contrast, in 40% of 10 months old ^*NY*^*Fcγ*${RIIb}_{B6}^{-/-}$ mice total IgG anti-nuclear Abs was significantly increased compared to C57BL/6 mice (110). But only five percent of ^*NY*^*Fcγ*${RIIb}_{B6}^{-/-}$ mice showed premature death whereas mortality was not increased in ^*Le*^*Fcγ*${RIIb}_{B6}^{-/-}$ mice although proteinuria and kidney pathology were significantly higher in these mice compared to C57BL/6 mice. The kidney phenotype in the absence of detectable ANA in ^*Le*^*Fcγ*${RIIb}_{B6}^{-/-}$ mice points to a protective role of FcγRIIb in the kidney, in the efferent phase, as has also been shown in a model of antibody-induced nephrotoxic nephritis (NTN) that will be discussed later (89).

**Table 2:** Disease phenotypes of *Fcγ*${RIIb}_{B6}^{-/-}$, C57BL/6 *Fcγ*${RIIb}_{129}^{-/-}$ *Slamf*_*B*6_ congenic, C57BL/6 *Slamf*_129_ congenic and the original *Fcγ*${RIIb}_{129}^{-/-}$ mice compared to WT C57BL/6 control mice at the age of 6–8 months.

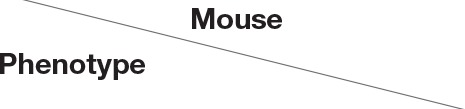	***^Le^Fcγ$RIIb_{B6}^{-/-\;}$^c^***	***C57BL6 Fcγ$RIIb_{129}^{-/-}$ Slamf_***B*6**_* Congenic^a^**	***^***NY***^Fcγ$RIIb_{B6}^{-/-\;}$^b^^,^^d^***	***C57BL/6 Slamf_129_* Congenic^a^^,^^b^**	***Fcγ$RIIb_{129}^{-/-}$^a^^,^^b^^,^^c^^,^^d^***
Increased IgM	n.d.	–^a^	n.d.	–^a^	+^a^
Increased IgG^a^	n.d.	+(♀)^a^	n.d.	–^a^	+ (♀ ♂)^a^
α-DNA	+ (♀) Total IgG Incidence 5%^c^	+(♀) IgG2c^a^	++ (♀) IgG2c^b^ Total IgG^d^	+++IgG2c/2b^b^(♀) IgG2c^a^	+++++++ (♀) IgG2c^a^^,^ ^b^ IgG2b^b^ Total IgG^a^^,^ ^d^
α-histone	– (♀) Total IgG^c^	n.d.	++ (♀) IgG2c^b^	+++IgG2c/2b^b^	+++++++ IgG2c/2b^b^ Total IgG^c^
α-nuclear	+ (♀) Total IgG^c^	+(♀) IgG2c^a^	++ (♀) IgG2c^b^ Total IgG Incidence 40%^d^	+++IgG2c^a^^,^ ^b^ IgG2b^b^	+++++++ (♀) IgG2c^a^^,^ ^b^ IgG2b^b^ Total IgG^a^^,^ ^d^
Kidney pathology	+ (♀)^c^	+ (♀)^a^	++^b^	–^a^^,^ ^b^	+++++^a^^,^ ^b^^,^ ^c^
IgG-IC deposition in glomeruli	+ (♀)*^c^*	+ (♀)*^a^*	++ (♀)^b^	+ (♀)^b^– (♀)^a^	++++ (♀ ♂)^a^^,^ ^b^^,^ ^c^
C3 deposition	+^c^	n.d.	–^b^	+^b^	++++^b^^,^ ^c^
Spleen	Slightly enlarged (♀)^c^	Slightly enlarged (♀)^a^	n.d.	Slightly enlarged (♀)^a^	Splenomegaly^a^^,^ ^b^^,^ ^c^
Spt-GC formation	n.d.	Normal(♀ ♂)^a^	Augmented + (♀)^b^	Augmented++ (♀)^a^^,^ ^b^	Augmented +++ (♀)^a^^,^ ^b^
% GC B cells of CD19^+^ splenic B cells	n.d.	No increase(♀)^a^	Increase +^b^	Increase ++(♀)^a^	Increase +++ (♀)^a^
Absolute numbers of splenic GC B cells	n.d.	No increase(♀)^a^	n.d.	Increase +*^(♀)^^a^*	Increase ++ (♀)^a^
Increased Mortality	–^c^	–^a^	+ 5%^d^	–^a^^,^ ^b^	Varies from 0%^a^ (and 22%^c^) to 60%^d^

a *Kanari et al. (98)*.

b *Soni et al. (163)*.

c *Boross et al. (109)*.

d *Li et al. (110)*.

*n.d., not determined*.

The production of autoantibodies by C57BL/6 mice in the absence of FcγRIIb suggests that FcγRIIb deficiency, besides modifying autoimmunity caused by other autoimmune susceptibility loci (e.g., *Slamf*_129_*, Yaa*), as discussed earlier, can result in loss of tolerance in the GC. However, it is tempting to speculate that the low titers of autoantibodies, that develop with low penetrance in *FcγRIIb* KO mice on a pure C57BL/6 background, reflect the natural occurring autoreactive B cells in the GC of a WT C57BL/6 mouse, as described earlier, that are prevented to enter the AFC pathway in the presence of FcγRIIb (178). There are indications that C57BL/6 mice are more autoimmune prone than BALB/c mice. For example, B cell receptor editing as a mechanism to maintain B cell tolerance is less effective in these mice compared to BALB/c mice (178).

The ^*NY*^*Fcγ*${RIIb}_{B6}^{-/-}$ mouse seems to exhibit a stronger disease phenotype than the ^*Le*^*Fcγ*${RIIb}_{B6}^{-/-}$ mouse (Table 2). There are several explanations for this discrepancy:

- The strains are generated with different ES cell lines. There might be relevant genomic differences between the C57BL/6 derived ES cell lines used. This question can be answered by sequencing the *FcγRIIb* flanking genomic regions in both mouse strains.
- The mice have been backcrossed several generations into different C57BL/6 mouse strains. There are substantial genetic variations between the different C57BL/6 strains used in different laboratories (184).
- Environmental factors (immune status, microbiome) play a role. The incidence of lethal disease in *Fcγ*${RIIb}_{129}^{-/-}$ mice varies between different laboratories from 0% to more than 60% (98, 109, 112, 173).
- Differences in the methods used to measure ANA. In the ^*Le*^*Fcγ*${RIIb}_{B6}^{-/-}$ mouse ANA have been measured only by ELISA of total IgG (109), whereas in the ^*NY*^*Fcγ*${RIIb}_{B6}^{-/-}$ mouse IgG2a and IgG2b have been measured combined with Hep-2 cell staining (163). However, a significant increase in total IgG anti-nuclear Abs compared to C57BL/6 has also been reported with the ^*NY*^*Fcγ*${RIIb}_{B6}^{-/-}$ mouse (110).

### The Individual Contribution of FcγRIIb Deficiency and Slamf_129_ to the Phenotype of the FcγRIIb KO Mouse on Mixed 129/C57BL/6 Background

Independently, in two different laboratories congenic C57BL6 *Slamf*_129_ mice have been generated. One was generated by intensive backcrossing of the original *Fcγ*${RIIb}_{129}^{-/-}$ mouse (161) into C57BL/6 background and selection for offspring in which the *Slamf* locus and the *FcγRIIb* KO allele had been segregated (98) resulting in two congenic strains called here as C57BL/6 *Slamf*_129_ congenic and C57BL/6 *Fcγ*${RIIb}_{129}^{-/-}$ *Slamf*_B6_ congenic, respectively. The other C57BL6 *Slamf*_129_ congenic mice were generated by a marker-assisted speed congenic approach (163) (Figure 1).

The development of autoimmunity was compared between C57BL/6 *Fcγ*${RIIb}_{129}^{-/-}$ *Slamf*_*B*6_ congenic, C57BL/6 *Slamf*_129_ congenic and the original *Fcγ*${RIIb}_{129}^{-/-}$ mice (98) or between C57BL/6 *Slamf*_129_ congenic, ^*NY*^*Fcγ*${RIIb}_{B6}^{-/-}$ and the original *Fcγ*${RIIb}_{129}^{-/-}$ mice (163). Both C57BL/6 *Fcγ*${RIIb}_{129}^{-/-}$ *Slamf*_*B*6_ congenic and C57BL/6 *Slamf*_129_ congenic mice developed very mild disease symptoms whereas the original *Fcγ*${RIIb}_{129}^{-/-}$ mice developed severe disease compared to WT C57BL/6 mice. Importantly, the phenotype of the C57BL/6 *Fcγ*${RIIb}_{129}^{-/-}$*Slamf*_B6_ congenic mouse strain confirmed mainly the phenotype of the ^*Le*^*Fcγ*${RIIb}_{B6}^{-/-}$ mouse [(98); Table 2] showing very low ANA titers and little kidney pathology compared to *Fcγ*${RIIb}_{129}^{-/-}$ mice.

The development of Spt-GC B cell and T_FH_ responses in C57BL/6 *Slamf*_129_ congenic, ^*NY*^*Fcγ*${RIIb}_{B6}^{-/-}$ and *Fcγ*${RIIb}_{129}^{-/-}$ mice were carefully compared [(163); Table 3]. C57Bl/6 *Slamf*_129_ congenic mice had significantly more GC B cells and T_FH_ and GC T_FH_ cells 12 days after immunization with OVA compared to WT C57BL/6 mice. B cells and DCs from *Slamf*_129_ congenic mice exhibited stronger antigen presentation in *in vitro* assays compared to B cells and DCs from WT C57BL/6 mice. By using a variety of *in vivo* and *in vitro* assays with naïve B cells it was found that B cell-intrinsic deficiency of FcγRIIb and expression of Slamf_129_ has no effect on proliferation but promotes differentiation of naïve B cells into GC B cells as indicated by increased expression of Aicda and GL-7. The percentage of apoptotic GC B cells was significantly lower in *Fcγ*${RIIb}_{129}^{-/-}$ mice compared to WT C57BL/6 mice whereas in C57BL/6 *Slamf*_129_ congenic and ^*NY*^*Fcγ*${RIIb}_{B6}^{-/-}$ mice this decrease was not significant. This suggests that FcγRIIb deficiency and Slamf_129_ act synergistically to increase the survival of GC B cells in *Fcγ*${RIIb}_{129}^{-/-}$ mice. Naïve and activated B cells from ^*NY*^*Fcγ*${RIIb}_{B6}^{-/-}$ and to a lower extent from C57BL/6 *Slamf*_129_ congenic mice showed an enhanced metabolic capacity compared to B cells from C57BL/6 mice. This enhancement was stronger in *Fcγ*${RIIb}_{129}^{-/-}$ mice.

**Table 3:** Characteristics of GC B and T cells in ^*NY*^*Fcγ*${RIIb}_{B6}^{-/-}$, C57BL/6 *Slamf*_129_ congenic, and the original *Fcγ*${RIIb}_{129}^{-/-}$ mice compared with WT C57BL/6 control mice.

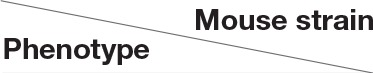	***Fcγ$RIIb_{129}^{-/-}$***	**C57BL/6 *Slamf_**129**_* congenic**	***^***NY***^Fcγ$RIIb_{B6}^{-/-\;}$***
Increase in frequency of B220^+^PNA^hi^ CD95^hi^ Spt-GC B cells	++++	++	+
Increase in Splenic GC size	+++	++	+
Increase in frequency of CD4^+^CXCR5^hi^PD-1^hi^ GC T_FH_ cells	+++	+	–
Increase in frequency of CD4^+^CXCR5^int^PD-1^int^ T_FH_ cells	+++	+	–
Increase in CD4^+^GL7^+^ GC T_FH_ cells	++	+	–
IL-21 expression in GC T_FH_ cells	++++	++	–
PD-1 expression in GC T_FH_ cells	++++	++	++
ICOS expression in GC T_FH_ cells	++	–	–
Increase in frequency of GC B cells upon antigenic stimulation	n.d.	+	–
Increase in frequency of GC T_FH_ cells upon antigenic stimulation	n.d.	+	–
MHC class II upregulation on GC B cells upon antigenic stimulation	n.d.	+	–
Decrease of caspase activity in DAPI^neg^B220^+^Fas^hi^PNA^hi^ GC B cells	++	+/–	+/–

*n.d., not determined (163)*.

Taken together these observations suggest that *Slamf*_129_ plays a predominant, and FcγRIIb deficiency a modest role in modulating the Spt-GC B cell and T_FH_ responses. Some of their functions are synergistic others mutually exclusive. GC T_FH_ cell responses are mainly affected by Slamf_129_ [(163); Table 3]. By using the experimental model of the V_H_ chain knockin strain HKIR mentioned earlier (179) it was demonstrated that B cell-specific expression of Slamf_129_ is necessary for the autoreactive B cells to expand in the GC confirming previous observations in C57BL/6 *Sle1* congenic mice (129).

The increased Spt-GC responses in ^*NY*^*Fcγ*${RIIb}_{B6}^{-/-}$ and C57BL/6 *Slamf*_129_ congenic mice were associated with the production of autoantibodies. However, the titers were much lower than in *Fcγ*${RIIb}_{129}^{-/-}$ mice which had also the strongest increase in Spt-GC responses. C57BL/6 *Slamf*_129_ congenic mice developed higher ANA titers than ^*NY*^*Fcγ*${RIIb}_{B6}^{-/-}$ mice, staining both cytoplasm and nucleus of Hep-2 cells, whereas sera from ^*NY*^*Fcγ*${RIIb}_{B6}^{-/-}$ mice show only cytoplasmic staining patterns (163) confirming previous results with the C57BL/6 *Fcγ*${RIIb}_{129}^{-/-}$ *Slamf*_*B*6_ congenic mouse strain (98). IgG2b and IgG2c ANA were significantly increased in C57BL/6 *Slamf*_129_ congenic mice whereas only IgG2c ANA were significantly increased in ^*NY*^*Fcγ*${RIIb}_{B6}^{-/-}$ mice. With an autoantigen array, it was shown that *Fcγ*${RIIb}_{129}^{-/-}$ mice develop high titers of IgG antibodies against a large variety of autoantigens. Several of these antibodies were also present in the serum of ^*NY*^*Fcγ*${RIIb}_{B6}^{-/-}$ mice but their titers were much lower than in *Fcγ*${RIIb}_{129}^{-/-}$ mice (163). Unfortunately, sera from C57BL/6 *Slamf*_129_ congenic mice were not tested in the autoantigen array.

Kidney pathology was absent (98) or very mild, with higher complement deposition than ^*NY*^*Fcγ*${RIIb}_{B6}^{-/-}$ mice (163), in C57BL/6 *Slamf*_129_ congenic mice, mild in ^*NY*^*Fcγ*${RIIb}_{B6}^{-/-}$ or C57BL/6 *Fcγ*${RIIb}_{129}^{-/-}$ *Slamf*_*B*6_ congenic mice with higher IgG deposition than in C57BL/6 *Slamf*_129_ congenic mice, and severe, with highest C3 and IgG deposition compared to the other genotypes, in *Fcγ*${RIIb}_{129}^{-/-}$ mice (98, 163). In conclusion, the deficiency of FcγRIIb together with the presence of Slamf_129_ results in a phenotype of the *Fcγ*${RIIb}_{129}^{-/-}$ mouse with increased Spt-GC B cell responses characterized by an increase of the following parameters: metabolic activity in B cells, differentiation of B cells into a GC B cell phenotype and GC B cell survival. This is associated with loss of immune tolerance resulting in ANA production and the development of severe lupus-like disease (163). However, the underlying cellular and molecular mechanisms of these associations are not well-understood and the subject of speculation and debate with respect to the role of FcγRIIb in GC (185). This can be illustrated with the surprising observation in the *FcγRIIb*_*NZB*_ KI mouse model mentioned earlier, in which FcγRIIb failed to be upregulated on activated and GC B cells resulting in enhanced early GC responses (11). Upon immunization, these KI mice showed an early and sustained increased affinity maturation of Ag-specific GC B cells. Previous models suggest that low expression of FcγRIIb reduces the BCR activation threshold resulting in less affinity maturation. However, an alternative explanation might be that low FcγRIIb expression increases the survival of bystander Ag non-specific GC B cells and, as a consequence, increases competition for T_FH_ help between Ag-specific and non-antigen specific B cells, resulting in increased affinity maturation (11).

### Cell-Type-Specific *FcγRIIb* KO Mouse Models

To determine on what B cell subset(s) and on what myeloid cells FcγRIIb might be involved in a checkpoint for immune tolerance, cell-type-specific *FcγRIIb*^−/−^ mice were generated, independently, in two different laboratories. Both the ^*Le*^*Fcγ*${RIIb}_{B6}^{-/-}$ and ^*NY*^*Fcγ*${RIIb}_{B6}^{-/-}$ mouse models, on a pure C57BL/6 background, were originally generated as floxed *FcγRIIb* mice (*Fcγ*${RIIb}_{B6}^{fl/fl}$) and subsequently crossed with a Cre deleter transgenic mouse to generate the germline *Fcγ*${RIIb}_{B6}^{-/-}$ mice discussed earlier. In addition, the *Fcγ*${RIIb}_{B6}^{fl/fl}$ mice were also crossed with a variety of cell type-specific Cre transgenic mice (Table 4) to generate cell-type-specific *Fcγ*${RIIb}_{B6}^{-/-}$ strains that were analyzed in the following models of diseases for which germline *FcγRIIb* KOs are highly susceptible: (a) the induced autoimmune diseases CIA, both on permissive (immunization with chicken collagen type II) and non-permissive (immunization with bovine collagen type II) background and (b) anti-glomerular basement membrane antibody (anti-GBM) disease, (c) the spontaneous autoimmune disease lupus-like disease and (d) the non-autoimmune disease antibody-induced NTN.

**Table 4:** Disease susceptibility of cell-type-specific *FcγRIIb* KO mice.

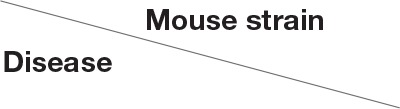	***CD19Cre: All B cells*^a^^,^^b^^,^^c^**	***LysMCre: Subset monocytes*^a^^,^^d^**	***cEBPαCre*: *pan-myeloid*^b^^,^^c^**	***CD11cCre*: *DCs*^c^^,^^d^**	***Mb1Cre*: *All B cells*^d^**	***Cg1Cre*: *GC and post GC B cells*^d^**
Non-permissive bCIA^c^^,^ ^d^	No increase	No increase *^*NY*^Fcγ${RIIb}_{B6}^{fl/fl}$* ^d^	n.d.	Increase*^*NY*^Fcγ${RIIb}_{B6}^{fl/fl}$* ^d^	Increase *^*NY*^Fcγ${RIIb}_{B6}^{fl/fl}$* ^d^	No increase*^*NY*^Fcγ${RIIb}_{B6}^{fl/fl}$* ^d^
Permissive cCIA^c^^,^ ^d^	No increase *^*Le*^Fcγ${RIIb}_{B6}^{fl/fl}$* ^c^	n.d.	Increase *^*Le*^Fcγ${RIIb}_{B6}^{fl/fl}$* ^c^	No increase*^*Le*^Fcγ${RIIb}_{B6}^{fl/fl}$* ^c^	n.d.	Increase similar to *^*NY*^Fcγ${RIIb}_{B6}^{-/-}$*^d^
KRN arthritis^d^	n.d.	Increase*^*NY*^Fcγ${RIIb}_{B6}^{fl/fl}$* ^d^	n.d.	n.d.	n.d.	n.d.
Anti-GBM disease^a^	No increase *^*Le*^Fcγ${RIIb}_{B6}^{fl/fl}$* ^a^	No increase*^*Le*^Fcγ${RIIb}_{B6}^{fl/fl}$* ^a^	n.d.	n.d.	n.d.	n.d.
Lupus-like disease^d^	n.d.	No ANA *^*NY*^Fcγ${RIIb}_{B6}^{fl/fl}$* ^d^	n.d.	No ANA*^*NY*^Fcγ${RIIb}_{B6}^{fl/fl}$* ^d^	No ANA *^*NY*^Fcγ${RIIb}_{B6}^{fl/fl}$* ^d^	ANAsimilar to *^*NY*^Fcγ${RIIb}_{B6}^{-/-}$*^d^
NTN^b^	No increase *^*Le*^Fcγ${RIIb}_{B6}^{fl/fl}$* ^b^	n.d.	Increase *^*Le*^Fcγ${RIIb}_{B6}^{fl/fl}$* ^b^	n.d.	n.d.	n.d.
Immunization^d^	n.d.	No increase in IgG response *^*NY*^Fcγ${RIIb}_{B6}^{fl/fl}$* ^d^	n.d.	No increase in IgG response *^*NY*^Fcγ${RIIb}_{B6}^{fl/fl}$* ^d^	Increased primary/secondary IgG response *^*NY*^Fcγ${RIIb}_{B6}^{fl/fl}$* ^d^	Increased secondary IgG response*^*NY*^Fcγ${RIIb}_{B6}^{fl/fl}$* ^d^

*Germline FcγRIIb KO mice showed increased susceptibility to all diseases listed in the table compared with C57BL/6 mice*.

a *Sharp et al. (186)*.

b *Sharp et al. (89)*.

c *Yilmaz-Elis et al. (187)*.

d *Li et al. (110)*.

*n.d., not determined*.

Deletion of *FcγRIIb* in all B cells of the ^*Le*^*Fcγ*${RIIb}_{B6}^{fl/fl}$ mouse by *CD19Cre* did not increase the susceptibility of this mouse for any of the mentioned disease models. Moreover, deletion of *FcγRIIb* on a subset of monocytes (*LysMCre*) had no effect on susceptibility for anti-GBM disease. Therefore, it was concluded that FcγRIIb deficiency on B cells or a subset of myeloid cells alone is not sufficient to increase susceptibility to anti-GBM (186). Only pan-myeloid deletion (*cEBP*α*Cre*) of FcγRIIb increased the susceptibility of ^*Le*^*Fcγ*${RIIb}_{B6}^{fl/fl}$ mice for CIA on the permissive background (187) and for the non-autoimmune disease NTN (89). These results suggest that for the protection against induced auto-Ab driven diseases, such as CIA, the role of FcγRIIb on B cells, as a checkpoint for immune tolerance, is less important than its role on myeloid effector cells, controlling downstream antibody effector mechanisms (187). However, it cannot be excluded that in the CIA model FcγRIIb on myeloid cells also plays a role in controlling the afferent phase of the disease, as was recently shown in *Yaa*^+^^*Le*^*Fcγ*${RIIb}_{B6}^{-/-}$ mice that will be discussed later (188).

In contrast to the results with ^*Le*^*Fcγ*${\text{IIb}}_{B6}^{fl/fl}$ mice, deletion of FcγRIIb in all B cells (*Mb1Cre*) or in GC and post GC B cells (*Cg1Cre*) in ^*NY*^*Fcγ*${RIIb}_{B6}^{fl/fl}$ mice resulted in increased susceptibility for CIA on the non-permissive background and permissive background, respectively. Moreover, susceptibility to CIA was also increased in DC-specific *CD11cCre*/^*NY*^*Fcγ*${RIIb}_{B6}^{fl/fl}$ mice indicating that FcγRIIb is involved in distinct immune tolerance controlling mechanisms (110). The reason for the discrepancy between the phenotypes of the B cell- and DC-specific ^*NY*^*Fcγ*${RIIb}_{B6}^{-/-}$ and ^*Le*^*Fcγ*${\text{IIb}}_{B6}^{-/-}$ mice is not known but, given the weak phenotype of the germline ^*Le*^*Fcγ*${RIIb}_{B6}^{-/-}$ mouse, most likely the phenotype of a single cell-type-specific ^*Le*^*Fcγ*${RIIb}_{B6}^{-/-}$ mouse is too weak to be detected with a small cohort of mice. Another partial explanation might be that the B-cell-specific Cre lines used are different. In addition, GC and post GC B cell (*Cg1Cre*) specific ^*NY*^*Fcγ*${RIIb}_{B6}^{-/-}$ mice developed spontaneously ANA, similar to ANA in germline ^*NY*^*Fcγ*${RIIb}_{B6}^{-/-}$ mice, whereas a deficiency in other cell types has no effect. This confirms previous results with transplantation of bone marrow from *Fcγ*${RIIb}_{129}^{-/-}$ mice that the role of FcγRIIb in the spontaneous development of ANA is B cell-specific (112) and suggests that FcγRIIb on GC or post GC B cells is a checkpoint for the maintenance of immune tolerance (110) (Table 4).

Upon immunization with the NP-CGG model antigen ^*NY*^*Fcγ*${RIIb}_{B6}^{-}$^/−^ and *Mb1Cre/*^*NY*^*Fcγ*${RIIb}_{B6}^{fl/fl}$ mice developed similar increased primary IgG NP-specific Ab responses compared to ^*NY*^*Fcγ*${RIIb}_{B6}^{fl/fl}$ mice and all other cell type-specific ^*NY*^*Fcγ*${RIIb}_{B6}^{-/-}$ mice. In contrast, secondary IgG Ab responses were increased in both *Mb1Cre/*^*NY*^*Fcγ*${RIIb}_{B6}^{fl/fl}$ and *Cg1Cre/*^*NY*^*Fcγ*${RIIb}_{B6}^{fl/fl}$ mice compared with ^*NY*^*Fcγ*${RIIb}_{B6}^{fl/fl}$ mice. This suggests that FcγRIIb is a B cell-intrinsic negative regulator of both primary and secondary IgG responses (110).

Although individually not sufficient to induce substantial autoimmunity, epistasis between the *Yaa* locus, the ^*Le*^*Fcγ*${RIIb}_{B6}^{-/-}$ alleles and the C57BL/6 genome results in severe lupus-like disease (109) (Figure 1). The cell-type-specific role of FcγRIIb in this genetic disease model was studied (188). The *Yaa*^+^/*CD19Cre/*^*Le*^*Fcγ*${RIIb}_{B6}^{fl/fl}$ mice developed milder lupus-like disease than *Yaa*^+^/^*Le*^*Fcγ*${RIIb}_{B6}^{-/-}$ mice similar to the disease in *Yaa*^+^/*C/EBP*α *Cre/*^*Le*^*Fcγ*${RIIb}_{B6}^{fl/fl}$ mice whereas *Yaa*^+^/*CD11cCre/*^*Le*^*Fcγ*${RIIb}_{B6}^{fl/fl}$ mice stayed disease free, like *Yaa*^+^/^*Le*^*Fcγ*${RIIb}_{B6}^{fl/fl}$ mice. This suggests that besides on B cells FcγRIIb on myeloid cells, but surprisingly not on DCs, contributes to the protection against spontaneous loss of immune tolerance in this mouse model. This confirms the observation with CIA in mice (110), discussed earlier, that FcγRIIb can be involved in different immune tolerance controlling mechanisms.

Strikingly, in the two strains with FcγRIIb deficient myeloid cells (*Yaa*^+^/^*Le*^*Fcγ*${RIIb}_{B6}^{-/-}$ and *Yaa*^+^/*C/EBP*α *Cre/*^*Le*^*Fcγ*${RIIb}_{B6}^{fl/fl}$) but not in the strain with B cell-specific FcγRIIb deficiency (*Yaa*^+^/*CD19Cre/*^*Le*^*Fcγ*${RIIb}_{B6}^{fl/fl}$) the frequency of peripheral Ly6C^−^, but not Ly6C^+^ monocytes was increased. Monocytosis, an FcRγ dependent expansion of the monocyte compartment consisting mainly of Ly6C^−^ monocytes, is associated with the development of lupus nephritis in *Yaa*^+^ lupus-prone mice. It has been reported that Ly6C^+^ monocytes mature in the circulation and are the precursors for Ly6C^−^ monocytes (189). Deficiency of FcγRIIb most likely accelerates the maturation of monocytes in *Yaa*^+^/^*Le*^*Fcγ*${RIIb}_{B6}^{-/-}$ mice. Compared to Ly6C^+^ monocytes, mature Ly6C^−^ monocytes express significantly higher B cell-stimulating cytokines such as BSF-3, IL-10, and IL-1β, DC markers including CD11c, CD83, Adamdec1, and the anti-apoptotic factors Bcl2 and Bcl6. This makes monocytes the most promising FcγRIIb expressing candidate myeloid cells to modulate B cell tolerance (188, 190). The transcriptome of Ly6C^−^ monocytes suggests that they are long-lived and committed to developing into DCs.

Whether this monocyte-dependent tolerance breaking mechanism is unique for *Yaa*^+^/*Fcγ*${RIIb}_{B6}^{-/-}$ mice is not known but it is striking that also in SLE patients the serum levels of anti-dsDNA Abs highly correlate with the percentage of non-classical monocytes (191). Like mouse Ly6C^−^ monocytes, the human counterpart CD14^low^CD16^+^ monocytes secrete high amounts of IL-1β in a TLR7-TLR8-MyD88–dependent manner (192).

## Concluding Remarks

Forward and reverse genetics have provided convincing evidence that FcγRIIb is an important autoimmune susceptibility gene, involved in the maintenance of peripheral tolerance both in human and mice. In humans, a number of GWAS studies showed an association between a SNP *(rs1050501*) in the *FCGR2B* gene, causing a missense mutation (*FCGR2B*^*T*232^) resulting in impaired FCGR2B function, and susceptibility to SLE. Meta-analyses confirmed that *FCGR2B*^*T*232^ homozygosity is one of the strongest associations in SLE. Association of *rs1050501*with RA has also been reported.

In mice, the situation is more diffuse. Analysis of a variety of C57BL/6 mice congenic for the NZW and NZB haplotypes of *FcγRIIb*, with decreased expression, did not reveal clear unambiguous results with respect to the contribution of these haplotypes to the autoimmune phenotypes of these mice. The mechanism by which natural FcγRIIb variants contribute to autoimmunity is not well-understood.

The first *FcγRIIb*^−/−^ mouse, generated by gene targeting in 129 derived ES cells and backcrossed into C57BL/6 background (*Fcγ*${RIIb}_{129}^{-/-}$ mice), exhibited a surprisingly strong spontaneous autoimmune phenotype suggesting that FcγRIIb deficiency initiates loss of immune tolerance. However, independent studies with *Fcγ*${RIIb}_{129}^{-/-}$ autoimmune *V*_*H*_ chain knockin mice pointed to a central role of FcγRIIb in a late immune tolerance checkpoint, that prevents autoimmunity by suppressing the production of autoreactive IgG from B cells, that escape negative selection in the GC and enter the AFC pathway. This should mean that FcγRIIb deficiency is mainly an amplifier of autoimmunity caused by other autoimmune susceptibility loci, rather than a primary initiator of the loss of immune tolerance. That was confirmed by the observation that *FcγRIIb*^−/−^ mice on a pure C57BL/6 background (*Fcγ*${RIIb}_{B6}^{-/-}$) have a much milder autoimmune phenotype than *Fcγ*${RIIb}_{129}^{-/-}$ mice but when backcrossed into a mouse strain carrying the autoimmune susceptibility *Yaa* locus succumb to lupus-like disease. The strong autoimmune phenotype of the *Fcγ*${RIIb}_{129}^{-/-}$ mouse could be explained by epistatic interactions between the C57BL/6 genome, the FcγRIIb KO allele and the 129 derived sequences (*Sle16*) flanking the *FcγRIIb* KO allele, containing the autoimmunity associated *Slamf*_129_ (haplotype 2) gene cluster.

Spt-GC B and T_FH_ cells are activated, modestly (mainly B cells) in *Fcγ*${RIIb}_{B6}^{-/-}$ mice, moderately in C57BL/6 *Slamf*_129_ congenic mice and strongly in *Fcγ*${RIIb}_{129}^{-/-}$ mice compared to Spt-GC B and T_FH_ cells in WT C57BL/6 mice. This was associated with a corresponding increase in ANA production, suggesting that FcγRIIb deficiency, besides enhancing autoimmunity caused by other autoimmune susceptibility loci, might play a modest role in the induction of the loss of immune tolerance in the GC, explaining the development with low penetrance of low ANA titers in *Fcγ*${RIIb}_{B6}^{-/-}$ mice. An alternative explanation is that the low ANA titers in *Fcγ*${RIIb}_{B6}^{-/-}$ mice reflect the natural background of autoreactive B cells in the GC that are prevented to enter the AFC pathway in the presence of FcγRIIb. The analysis of the development of self-reactive GC B cells and plasma cells by large scale Ig cloning from single isolated B cells, as performed with *Fcγ*${RIIb}_{129}^{-/-}$ mice, should be repeated in *Fcγ*${RIIb}_{B6}^{-/-}$ mice, to determine how FcγRIIb deficiency influences the frequency at which autoreactive ANA-expressing B cells participate in GC reactions, and develop in plasma cells, under physiological conditions, without the confounding effect of Slamf_129_ expression.

Studies with cell-type-specific FcγRIIb deficient mice revealed that besides on B cells, FcγRIIb on DCs and monocytes can also contribute to the maintenance of immune tolerance, indicating that FcγRIIb is involved in different immune tolerance maintaining mechanisms. Series of observations suggest that on B cells impaired FcγRIIb function effects not only antibody titers but also affinity maturation and memory responses of B cells and plasma cell homeostasis associated with an increase in the production of autoantibodies. However, the underlying cellular and molecular mechanisms are not well-understood. Most likely new model systems including adoptive cell transfer and tools such as cell type-specific KO mice, to study the GC reaction, are required to answer these questions.

## Author Contributions

All authors listed have made a substantial, direct and intellectual contribution to the work, and approved it for publication.

### Conflict of Interest

The authors declare that the research was conducted in the absence of any commercial or financial relationships that could be construed as a potential conflict of interest.

## References

[1] Takai T Li M Sylvestre D Clynes R Ravetch JV FcRγ chain deletion results in pleiotrophic effector cell defects. Cell. (1994) 76:519–29. 10.1016/0092-8674(94)90115-5 8313472

[2] Nimmerjahn F Ravetch JV. Fc-receptors as regulators of immunity. Adv Immunol. (2007) 96:179–204. 10.1016/S0065-2776(07)96005-8 17981207

[3] Getahun A Cambier JC. Of ITIMs, ITAMs, and ITAMis: revisiting immunoglobulin Fc receptor signaling. Immunol Rev. (2015) 268:66–73. 10.1111/imr.12336 26497513 PMC4621791

[4] Tsao BP Cantor RM Kalunian KC Chen CJ Badsha H Singh R. Evidence for linkage of a candidate chromosome 1 region to human systemic lupus erythematosus. J Clin Invest. (1997) 99:725–31. 10.1172/JCI119217 9045876 PMC507856

[5] Daeron M Latour S Malbec O Espinosa E Pina P Pasmans S The same tyrosine-based inhibition motif, in the intracytoplasmic domain of Fcγ RIIB, regulates negatively BCR-, TCR-, and FcR-dependent cell activation. Immunity. (1995) 3:635–46. 10.1016/1074-7613(95)90134-5 7584153

[6] Daeron M Jaeger S Du Pasquier L Vivier E. Immunoreceptor tyrosine-based inhibition motifs: a quest in the past and future. Immunol Rev. (2008) 224:11–43. 10.1111/j.1600-065X.2008.00666.x 18759918

[7] Daeron M. Fc receptor biology. Annu Rev Immunol. (1997) 15:203–34. 10.1146/annurev.immunol.15.1.203 9143687

[8] Miettinen HM Rose JK Mellman I. Fc receptor isoforms exhibit distinct abilities for coated pit localization as a result of cytoplasmic domain heterogeneity. Cell. (1989) 58:317–27. 10.1016/0092-8674(89)90846-5 2568890

[9] Miettinen HM Matter K Hunziker W Rose JK Mellman I. Fc receptor endocytosis is controlled by a cytoplasmic domain determinant that actively prevents coated pit localization. J Cell Biol. (1992) 116:875–88. 10.1083/jcb.116.4.875 1734021 PMC2289334

[10] Xiang Z Cutler AJ Brownlie RJ Fairfax K Lawlor KE Severinson E. FcγRIIb controls bone marrow plasma cell persistence and apoptosis. Nat Immunol. (2007) 8:419–29. 10.1038/ni1440 17322888

[11] Espéli M Clatworthy MR Bökers S Lawlor KE Cutler AJ Köntgen F. Analysis of a wild mouse promoter variant reveals a novel role for FcγRIIb in the control of the germinal center and autoimmunity. J Exp Med. (2012) 209:2307–19. 10.1084/jem.20121752 23109709 PMC3501356

[12] Amezcua Vesely MC Schwartz M Bermejo DA Montes CL Cautivo KM Kalergis AM FcγRIIb and BAFF differentially regulate peritoneal B1 cell survival. J Immunol. (2012) 88:4792–800. 10.4049/jimmunol.1102070 PMC3361506 22516957

[13] Rudge EU Cutler AJ Pritchard NR Smith KG Interleukin 4 reduces expression of inhibitory receptors on B cells and abolishes CD22 and FcγRII-mediated B cell suppression. J Exp Med. (2002) 195:1079–85. 10.1084/jem.20011435 11956299 PMC2193690

[14] Nimmerjahn F Ravetch JV. Fcgγ receptors as regulators of immune responses. Nat Rev Immunol. (2008) 8:34–47 10.1038/nri2206 18064051

[15] Starbeck-Miller GR Badovinac VP Barber DL Harty JT. Cutting edge: expression of FcγRIIB tempers memory CD8 T cell function *in vivo*. J Immunol. (2014) 192:35–9. 10.4049/jimmunol.1302232 24285839 PMC3874719

[16] Guilliams M Bruhns P Saeys Y Hammad H Lambrecht BN. The function of Fcγ receptors in dendritic cells and macrophages. Nat Rev Immunol. (2014) 14:94–108. 10.1038/nri3582 24445665

[17] Shushakova N Skokowa J Schulman J Baumann U Zwirner J Schmidt RE. C5a anaphylatoxin is a major regulator of activating versus inhibitory FcγRs in immune complex-induced lung disease. J Clin Invest. (2002) 110:1823–30. 10.1172/JCI200216577 12488432 PMC151656

[18] Skokowa J Ali SR Felda O Kumar V Konrad S Shushakova N Macrophages induce the inflammatory response in the pulmonary Arthus reaction through Gαi2 activation that controls C5aR and Fc receptor cooperation. J Immunol. (2005) 174:3041–50. 10.4049/jimmunol.174.5.3041 15728518

[19] Snapper CM Hooley JJ Atasoy U Finkelman FD Paul WE Differential regulation of murine B cell FcγRII expression by CD4+ T helper subsets. J Immunol. (1989) 143:2133–41. 2528589

[20] Liu Y Gao X Masuda E Redecha PB Blank MC Pricop L. Regulated expression of FcγR in human dendritic cells controls cross-presentation of antigen-antibody complexes. J Immunol. (2006) 177:8440–7. 10.4049/jimmunol.177.12.8440 17142741

[21] Tridandapani S Siefker K Teillaud JL Carter JE Wewers MD Anderson CL Regulated expression and inhibitory function of FcγRIIb in human monocytic cells. J Biol Chem. (2002) 277:5082–9. 10.1074/jbc.M110277200 11741917

[22] Tridandapani S Wardrop R Baran CP Wang Y Opalek JM Caligiuri MA TGF-β1 Suppresses Myeloid Fcγ Receptor Function by Regulating the Expression and Function of the Common γ-Subunit. J Immunol. (2003) 170:4572–7. 10.4049/jimmunol.170.9.4572 12707335

[23] Pricop L Redecha P Teillaud JL Frey J Fridman WH Sautes-Fridman C Differential modulation of stimulatory and inhibitory Fcγ receptors on human monocytes by Th1 and Th2 cytokines. J Immunol. (2001) 166:531–7. 10.4049/jimmunol.166.1.531 11123333

[24] Tutt AL James S Laversin SA Tipton TR Ashton-Key M French RR. Development and characterization of monoclonal antibodies specific for mouse and human Fcγ receptors. J Immunol. (2015) 195:5503–16. 10.4049/jimmunol.1402988 26512139

[25] Ganesan LP Kim J Wu Y Mohanty S Phillips GS Birmingham DJ. FcγRIIb on liver sinusoidal endothelium clears small immune complexes. J Immunol. (2012) 189:4981–8. 10.4049/jimmunol.1202017 23053513 PMC4381350

[26] Anderson CL Ganesan LP Robinson JM. The biology of the classical Fcγ receptors in non-hematopoietic cells. Immunol Rev. (2015) 268:236–40. 10.1111/imr.12335 26497524

[27] Radeke HH Janssen-Graalfs I Sowa EN Chouchakova N Skokowa J Loscher F. Opposite regulation of type II and III receptors for immunoglobulin G in mouse glomerular mesangial cells and in the induction of anti-glomerular basement membrane (GBM) nephritis. J Biol Chem. (2002) 277:27535–44. 10.1074/jbc.M200419200 11983693

[28] Ono M Bolland S Tempst P Ravetch JV. Role of the inositol phosphatase SHIP in negative regulation of the immune system by the receptor Fc(γ)RIIB. Nature. (1996) 383:263–6. 10.1038/383263a0 8805703

[29] Karsten CM Pandey MK Figge J Kilchenstein R Taylor PR Rosas M. Anti-inflammatory activity of IgG1 mediated by Fc galactosylation and association of FcγRIIB and dectin-1. Nat Med. (2012) 18:1401–6. 10.1038/nm.2862 22922409 PMC3492054

[30] Phillips NE Parker DC. Cross-linking of B lymphocyte Fcγ receptors and membrane immunoglobulin inhibits anti-immunoglobulin-induced blastogenesis. J Immunol. (1984) 132:627–32. 6228594

[31] Phillips NE Parker DC Subclass specificity of Fcγ receptor-mediated inhibition of mouse B cell activation. J Immunol. (1985) 134:2835–8. 3156919

[32] Coggeshall KM Inhibitory signaling by B cell FcγRIIb. Curr Opin Immunol. (1998) 10:306–12. 10.1016/S0952-7915(98)80169-6 9638367

[33] Pearse RN Kawabe T Bolland S Guinamard R Kurosaki T Ravetch JV SHIP recruitment attenuates FcγRIIB-induced B cell apoptosis. Immunity. (1999) 10:753–60. 10.1016/S1074-7613(00)80074-6 10403650

[34] Ashman RF Peckham D Stunz LL. Fc receptor off-signal in the B cell involves apoptosis. J Immunol. (1996) 157:5–11. 8683155

[35] Daeron M Malbec O Latour S Arock M Fridman WH. Regulation of high-affinity IgE receptor-mediated mast cell activation by murine low-affinity IgG receptors. J Clin Invest. (1995) 95:577–85. 10.1172/JCI117701 7860741 PMC295517

[36] Clynes R Dumitru C Ravetch JV. Uncoupling of immune complex formation and kidney damage in autoimmune glomerulonephritis. Science. (1998) 279:1052–4 10.1126/science.279.5353.1052 9461440

[37] Steinman RM Hawiger D Liu K Bonifaz L Bonnyay D Mahnke K. Dendritic cell function *in vivo* during the steady state: a role in peripheral tolerance. Ann N Y Acad Sci. (2003) 987:15–25. 10.1111/j.1749-6632.2003.tb06029.x 12727620

[38] Steinman RM Hawiger D Nussenzweig MC. Tolerogenic dendritic cells. Annu Rev Immunol. (2003) 21:685–711. 10.1146/annurev.immunol.21.120601.141040 12615891

[39] Banchereau J Steinman RM. Dendritic cells and the control of immunity. Nature. (1998) 392:245–52. 10.1038/32588 9521319

[40] Merad M Sathe P Helft J Miller J Mortha A. The dendritic cell lineage: ontogeny and function of dendritic cells and their subsets in the steady-state and the inflamed setting. Annu Rev Immunol. (2013) 31:563–604. 10.1146/annurev-immunol-020711-074950 23516985 PMC3853342

[41] Eisenbarth SC. Dendritic cell subsets in T cell programming: location dictates function. Nat Rev Immunol. (2019) 19:89–103. 10.1038/s41577-018-0088-1 30464294 PMC7755085

[42] Heyman B. Antibodies as natural adjuvants. Curr Top Microbiol Immunol. (2014) 382:201–19. 10.1007/978-3-319-07911-0_9 25116101

[43] Regnault A Lankar D Lacabanne V Rodriguez A Thery C Rescigno M. Fcγ receptor-mediated induction of dendritic cell maturation and major histocompatibility complex class I-restricted antigen presentation after immune complex internalization. J Exp Med. (1999) 189:371–80. 10.1084/jem.189.2.371 9892619 PMC2192989

[44] Sedlik C Orbach D Veron P Schweighoffer E Colucci F Gamberale R. A critical role for Syk protein tyrosine kinase in Fc receptor-mediated antigen presentation and induction of dendritic cell maturation. J Immunol. (2003) 170:846–52. 10.4049/jimmunol.170.2.846 12517949

[45] Herrada AA Contreras FJ Tobar JA Pacheco R Kalergis AM. Immune complex-induced enhancement of bacterial antigen presentation requires Fcγ receptor III expression on dendritic cells. Proc Natl Acad Sci USA. (2007) 104:13402–7. 10.1073/pnas.0700999104 17679697 PMC1948949

[46] Yada A Ebihara S Matsumura K Endo S Maeda T Nakamura A. Accelerated antigen presentation and elicitation of humoral response *in vivo* by FcγRIIB- and FcγRI/III-mediated immune complex uptake. Cell Immunol. (2003) 225:21–32. 10.1016/j.cellimm.2003.09.008 14643301

[47] Kalergis AM Ravetch JV. Inducing tumor immunity through the selective engagement of activating Fcγ receptors on dendritic cells. J Exp Med. (2002) 195:1653–9. 10.1084/jem.20020338 12070293 PMC2193555

[48] Tobar JA González PA Kalergis AM Salmonella escape from antigen presentation can be overcome by targeting bacteria to Fcγ receptors on dendritic cells. J Immunol. (2004) 173:4058–65. 10.4049/jimmunol.173.6.4058 15356155

[49] Schuurhuis DH van Montfoort N Ioan-Facsinay A Jiawan R Camps M Nouta J. Immune complex-loaded dendritic cells are superior to soluble immune complexes as antitumor vaccine. J Immunol. (2006) 176:4573–80. 10.4049/jimmunol.176.8.4573 16585547

[50] Boross P van Montfoort N Stapels DA van der Poel CE Bertens C Meeldijk J. FcRγ-chain ITAM signaling is critically required for cross-presentation of soluble antibody-antigen complexes by dendritic cells. J Immunol. (2014) 193:5506–14. 10.4049/jimmunol.1302012 25355925

[51] Schuurhuis DH Ioan-Facsinay A Nagelkerken B van Schip JJ Sedlik C Melief CJ. Antigen-antibody immune complexes empower dendritic cells to efficiently prime specific CD8+ CTL responses *in vivo*. J Immunol. (2002) 168:2240–6. 10.4049/jimmunol.168.5.2240 11859111

[52] van Montfoort N t Hoen PA Mangsbo SM Camps MG Boross P Melief CJ. Fcγ receptor IIb strongly regulates Fcγ receptor-facilitated T cell activation by dendritic cells. J Immunol. (2012) 189:92–101. 10.4049/jimmunol.1103703 22649202

[53] Rafiq K Bergtold A Clynes R. Immune complex-mediated antigen presentation induces tumor immunity. J Clin Invest. (2002) 110:71–9. 10.1172/JCI15640 12093890 PMC151032

[54] Desai DD Harbers SO Flores M Colonna L Downie MP Bergtold A Fcγ receptor IIB on dendritic cells enforces peripheral tolerance by inhibiting effector T cell responses. J Immunol. (2007) 178:6217–26. 10.4049/jimmunol.178.10.6217 17475849

[55] Fransen MF Benonisson H van Maren WW Sow HS Breukel C Linssen MM. Restricted role for FcγR in the regulation of adaptive immunity. J Immunol. (2018) 200:2615–26. 10.4049/jimmunol.1700429 29523656 PMC5896742

[56] Ho NI Camps MGM de Haas EFE Trouw LA Verbeek JS Ossendorp F. C1q-dependent dendritic cell cross-presentation of *in vivo*-formed antigen-antibody complexes. J Immunol. (2017) 198:4235–43. 10.4049/jimmunol.1602169 28432146

[57] den Haan JM Bevan MJ. Constitutive versus activation-dependent cross-presentation of immune complexes by CD8(+) and CD8(-) dendritic cells *in vivo*. J Exp Med. (2002) 196:817–27. 10.1084/jem.20020295 12235214 PMC2194052

[58] Dhodapkar KM Kaufman JL Ehlers M Banerjee DK Bonvini E Koenig S. Selective blockade of inhibitory Fcγ receptor enables human dendritic cell maturation with IL-12p70 production and immunity to antibody-coated tumor cells. ProcNatlAcadSci USA. (2005) 102:2910–5. 10.1073/pnas.0500014102 15703291 PMC549508

[59] van Montfoort N Camps MG Khan S Filippov DV Weterings JJ Griffith JM. Antigen storage compartments in mature dendritic cells facilitate prolonged cytotoxic T lymphocyte cross-priming capacity. ProcNatlAcadSci USA. (2009) 106:6730–5. 10.1073/pnas.0900969106 19346487 PMC2672553

[60] Garcia De Vinuesa C Gulbranson-Judge A Khan M O'Leary P Cascalho M Wabl M. Dendritic cells associated with plasmablast survival. Eur J Immunol. (1999) 29:3712–21. 10.1002/(SICI)1521-4141(199911)29:11<3712::AID-IMMU3712>3.3.CO;2-G 10556827

[61] Balazs M Martin F Zhou T Kearney J. Blood dendritic cells interact with splenic marginal zone B cells to initiate T-independent immune responses. Immunity. (2002) 17:341–52. 10.1016/S1074-7613(02)00389-8 12354386

[62] Bergtold A Desai DD Gavhane A Clynes R. Cell surface recycling of internalized antigen permits dendritic cell priming of B cells. Immunity. (2005) 23:503–14. 10.1016/j.immuni.2005.09.013 16286018

[63] Gilliet M Cao W Liu YJ. Plasmacytoid dendritic cells: sensing nucleic acids in viral infection and autoimmune diseases. Nat Rev Immunol. (2008) 8:594–606. 10.1038/nri2358 18641647

[64] Reizis B. Intracellular pathogens and CD8(+) dendritic cells: dangerous liaisons. Immunity. (2011) 35:153–5. 10.1016/j.immuni.2011.08.003 21867923

[65] Goubier A Dubois B Gheit H Joubert G Villard-Truc F Asselin-Paturel C. Plasmacytoid dendritic cells mediate oral tolerance. Immunity. (2008) 29:464–75. 10.1016/j.immuni.2008.06.017 18789731 PMC3545652

[66] Irla M Kupfer N Suter T Lissilaa R Benkhoucha M Skupsky J. MHC class II-restricted antigen presentation by plasmacytoid dendritic cells inhibits T cell-mediated autoimmunity. J Exp Med. (2010) 207:1891–905. 10.1084/jem.20092627 20696698 PMC2931160

[67] Flores MD Desai DD Downie M Liang B Reilly MP McKenzie SE. Dominant expression of the inhibitory FcγRIIB prevents antigen presentation by murine plasmacytoid dendritic cells. J Immunol. (2009) 183:7129–39. 10.4049/jimmunol.0901169 19917701

[68] Bjorck P Beilhack A Herman EI Negrin RS Engleman EG. Plasmacytoid dendritic cells take up opsonized antigen leading to CD4+ and CD8+ T cell activation *in vivo*. J Immunol. (2008) 181:3811–7. 10.4049/jimmunol.181.6.3811 18768834 PMC2884144

[69] Benitez-Ribas D Adema GJ Winkels G Klasen IS Punt CJ Figdor CG Plasmacytoid dendritic cells of melanoma patients present exogenous proteins to CD4_ T cells after FcγRII-mediated uptake. J Exp Med. (2006) 203:1629–35. 10.1084/jem.20052364 16785312 PMC2118356

[70] Vollmer J Tluk S Schmitz C Hamm S Jurk M Forsbach A. Immune stimulation mediated by autoantigen binding sites within small nuclear RNAs involves Toll-like receptors 7 and 8. J Exp Med. (2005) 202:1575–85. 10.1084/jem.20051696 16330816 PMC2213330

[71] Ronnblom L Alm GV Eloranta ML. Type I interferon and lupus. Curr Opin Rheumatol. (2009) 21:471–7. 10.1097/BOR.0b013e32832e089e 19525849

[72] Means TK Latz E Hayashi F Murali MR Golenbock DT Luster AD. Human lupus autoantibody-DNA complexes activate DCs through the cooperation of CD32 and TLR9. J Clin Invest. (2005) 115:407–17. 10.1172/JCI200523025 15668740 PMC544604

[73] Nemazee D. Mechanisms of central tolerance for B cells. Nat Rev Immunol. (2017) 17:281–94. 10.1038/nri.2017.19 28368006 PMC5623591

[74] Cooke MP Heath AW Shokat KM Zeng Y Finkelman FD Linsley PS. Immunoglobulin signal transduction guides the specificity of B cell-T cell interactions and is blocked in tolerant self-reactive B cells. J Exp Med. (1994) 179:425–38. 10.1084/jem.179.2.425 8294858 PMC2191355

[75] Healy JI Dolmetsch RE Timmerman LA Cyster JG Thomas ML Crabtree GR. Different nuclear signals are activated by the B cell receptor during positive versus negative signaling. Immunity. (1997) 6:419–28. 10.1016/S1074-7613(00)80285-X 9133421

[76] Goodnow CC Crosbie J Jorgensen H Brink RA Basten A. Induction of self-tolerance in mature peripheral B lymphocytes. Nature. (1989) 342:385–91. 10.1038/342385a0 2586609

[77] Brink R Phan TG. Self-reactive B cells in the germinal center reaction. Annu Rev Immunol. (2018) 36:339–57. 10.1146/annurev-immunol-051116-052510 29356584

[78] Akkaraju S Canaan K Goodnow CC. (1997). Self-reactive B cells are not eliminated or inactivated by autoantigen expressed on thyroid epithelial cells. J.Exp.Med. 186, 2005–2012 10.1084/jem.186.12.2005 9396769 PMC2199176

[79] Aplin BD Keech CL de Kauwe AL Gordon TP Cavill D McCluskey J. Tolerance through indifference: autoreactive B cells to the nuclear antigen La show no evidence of tolerance in a transgenic model. J Immunol. (2003) 171:5890–900. 10.4049/jimmunol.171.11.5890 14634099

[80] El Shikh ME El Sayed RM Sukumar S Szakal AK Tew JG. Activation of B cells by antigens on follicular dendritic cells. Trends Immunol. (2010) 31:205–11. 10.1016/j.it.2010.03.002 20418164 PMC2886728

[81] Chan TD Wood K Hermes JR Butt D Jolly CJ Basten A. Elimination of germinal-center-derived self-reactive B cells is governed by the location and concentration of self-antigen. Immunity. (2012) 37:893–904. 10.1016/j.immuni.2012.07.017 23142780

[82] Krautler NJ Suan D Butt D Bourne K Hermes JR Chan TD. Differentiation of germinal center B cells into plasma cells is initiated by high-affinity antigen and completed by Tfh cells. J Exp Med. (2017) 214:1259–67. 10.1084/jem.20161533 28363897 PMC5413338

[83] Heesters BA van der Poel CE Das A Carroll MC. Antigen presentation to B cells. Trends Immunol. (2016) 37:844–54. 10.1016/j.it.2016.10.003 27793570

[84] Tew JG Wu J Fakher M Szakal AK Qin D. Follicular dendritic cells: beyond the necessity of T cell help. Trends Immunol. (2001) 22:361–7. 10.1016/S1471-4906(01)01942-1 11429319

[85] Ravetch JV Lanier LL. Immune inhibitory receptors. Science. (2000) 290:84–9. 10.1126/science.290.5489.84 11021804

[86] Ravetch JV Bolland S. IgG Fc receptors. Annu Rev Immunol. (2001) 19:275–90. 10.1146/annurev.immunol.19.1.275 11244038

[87] Ono M Okada H Bolland S Yanagi S Kurosaki T Ravetch JV. Deletion of SHIP or SHP-1 reveals two distinct pathways for inhibitory signaling. Cell. (1997) 90:293–301. 10.1016/S0092-8674(00)80337-2 9244303

[88] Tzeng SJ Bolland S Inabe K Kurosaki T Pierce SK. The B cell inhibitory Fc receptor triggers apoptosis by a novel c-Abl family kinase-dependent pathway. J Biol Chem. (2005) 280:35247–54. 10.1074/jbc.M505308200 16115887

[89] Sharp PE Martin-Ramirez J Mangsbo SM Boross P Pusey CD Touw IP. FcγRIIb on myeloid cells and intrinsic renal cells rather than B cells protects from nephrotoxic nephritis. J Immunol. (2013) 190:340–8. 10.4049/jimmunol.1202250 23203925

[90] Shirai T Hirose S Okada T Nishimura H Immunology and immunopathology of the autoimmune disease of NZB and related mouse strains. In: Rihova EB Vetvicka V, editors. Immunological Disorders in Mice. Boca Raton, FL: CRC Press, Inc (1991) 95–136.

[91] Reininger L Radaszkiewicz T Kosco M Melchers F Rolink AG. Development of autoimmune disease in SCID mice populated with long-term “*in vitro*” proliferating (NZB x NZW)F1 pre-B cells. J Exp Med. (1992) 176:1343–53. 10.1084/jem.176.5.1343 1402680 PMC2119422

[92] Reininger L Winkler TH Kalberer CP Jourdan M Melchers F Rolink AG. Intrinsic B cell defects in NZB and NZW mice contribute to systemic lupus erythematosus in (NZB x NZW)F1 mice. J Exp Med. (1996) 184:853–61. 10.1084/jem.184.3.853 9064345 PMC2192772

[93] Helyer BJ Howie JB Renal disease associated with positive lupus erythematosus tests in a cross-bred strain of mice. Nature. (1963) 12:197 10.1038/197197a0 13953664

[94] Theofilopoulos AN Dixon FJ. Murine models of systemic lupus erythematosus. Adv Immunol. (1985) 37:269–390. 10.1016/S0065-2776(08)60342-9 3890479

[95] Rudofsky UH Evans BD Balaban SL Mottironi VD Gabrielsen AE. Differences in expression of lupus nephritis in New Zealand mixed H-2z homozygous inbred strains of mice derived from New Zealand black and New Zealand white mice. Origins and initial characterization. Lab Invest. (1993) 68:419–26. 8479150

[96] Morel L Rudofsky UH Longmate JA Schiffenbauer J Wakeland EK. Polygenic control of susceptibility to murine systemic lupus erythematosus. Immunity. (1994) 1:219–29. 10.1016/1074-7613(94)90100-7 7889410

[97] Morel L Wakeland EK. Lessons from the NZM2410 model and related strains. Int Rev Immunol. (2000) 19:423–46. 10.3109/08830180009055506 11016426

[98] Kanari Y Sugahara-Tobinai A Takahashi H Inui M Nakamura A Hirose S Dichotomy in FcγRIIB deficiency and autoimmune-prone SLAM haplotype clarifies the roles of the Fc receptor in development of autoantibodies and glomerulonephritis. BMC Immunol. (2014) 24:47 10.1186/s12865-014-0047-y PMC4209029 25339546

[99] Mohan C Alas E Morel L Yang P Wakeland EK. Genetic dissection of SLE pathogenesis. Sle1 on murine chromosome 1 leads to a selective loss of tolerance to H2A/H2B/DNA subnucleosomes. J Clin Invest. (1998) 101:1362–72. 9502778 10.1172/JCI728 PMC508691

[100] Morel L Croker BP Blenman KR Mohan C Huang G Gilkeson G. Genetic reconstitution of systemic lupus erythematosus immunopathology with polycongenic murine strains. Proc Natl Acad Sci USA. (2000) 97:6670–5. 10.1073/pnas.97.12.6670 10841565 PMC18697

[101] Sobel ES Mohan C Morel L Schiffenbauer J Wakeland EK. Genetic dissection of SLE pathogenesis: adoptive transfer of Sle1 mediates the loss of tolerance by bone marrow-derived B cells. J Immunol. (1999) 162:2415–21. 9973523

[102] Sobel ES Satoh M Chen Y Wakeland EK Morel L. The major murine systemic lupus erythematosus susceptibility locus Sle1 results in abnormal functions of both B and T cells. J Immunol. (2002) 169:2694–700. 10.4049/jimmunol.169.5.2694 12193743

[103] Jiang Y Hirose S Abe M Sanokawa-Akakura R Ohtsuji M Mi X. Polymorphisms in IgG Fc receptor IIB regulatory regions associated with autoimmune susceptibility. Immunogenetics. (2000) 51:429–35. 10.1007/s002510050641 10866109

[104] Luan JJ Monteiro RC Sautes C Fluteau G Eloy L Fridman WH Defective FcγRII gene expression in macrophages of NOD mice: genetic linkage with up-regulation of IgG1 and IgG2b in serum. J Immunol. (1996) 157:4707–16. 8906852

[105] Pritchard NR Cutler AJ Uribe S Chadban SJ Morley BJ Smith KG. Autoimmune-prone mice share a promoter haplotype associated with reduced expression and function of the Fc receptor FcγRII. Curr Biol. (2000) 10:227–30. 10.1016/S0960-9822(00)00344-4 10704418

[106] Rahman ZS Manser T Failed up-regulation of the inhibitory IgG Fc receptor Fcγ RIIB on germinal center B cells in autoimmune-prone mice is not associated with deletion polymorphisms in the promoter region of the FcγRIIB gene. J Immunol. (2005) 175:1440–9. 10.4049/jimmunol.175.3.1440 16034080

[107] Xiu Y Nakamura K Abe M Li N Wen XS Jiang Y. Transcriptional regulation of Fcgr2b gene by polymorphic promoter region and its contribution to humoral immune responses. J Immunol. (2002) 169:4340–6. 10.4049/jimmunol.169.8.4340 12370366

[108] Rahman ZS Niu H Perry D Wakeland E Manser T Morel L. Expression of the autoimmune Fcgr2b NZW allele fails to be upregulated in germinal center B cells and is associated with increased IgG production. Genes Immun. (2007) 8:604–12. 10.1038/sj.gene.6364423 17713556

[109] Boross P Arandhara VL Martin-Ramirez J Santiago-Raber ML Carlucci F Flierman R. The inhibiting Fc receptor for IgG, FcγRIIB, is a modifier of autoimmune susceptibility. J Immunol. (2011) 187:1304–13. 10.4049/jimmunol.1101194 21724994

[110] Li F Smith P Ravetch JV. Inhibitory Fcγ receptor is required for the maintenance of tolerance through distinct mechanisms. J Immunol. (2014) 192:3021–8. 10.4049/jimmunol.1302934 24563255 PMC3967505

[111] Bygrave AE Rose KL Cortes-Hernandez J Warren J Rigby RJ Cook HT. Spontaneous autoimmunity in 129 and C57BL/6 mice-implications for autoimmunity described in gene-targeted mice. PLoS Biol. (2004) 2:E243. 10.1371/journal.pbio.0020243 15314659 PMC509305

[112] Bolland S Ravetch JV. Spontaneous autoimmune disease in Fc(γ)RIIB-deficient mice results from strain-specific epistasis. Immunity. (2000) 13:277–85. 10.1016/S1074-7613(00)00027-3 10981970

[113] Bolland S Yim YS Tus K Wakeland EK Ravetch JV. Genetic modifiers of systemic lupus erythematosus in FcγRIIB^−/−^ mice. J Exp Med. (2002) 195:1167–74. 10.1084/jem.20020165 11994421 PMC2193704

[114] Jorgensen TN Alfaro J Enriquez HL Jiang C Loo WM Atencio SA. Development of Murine Lupus involves the combined genetic contribution of the *SLAM* and *Fc**γ**R* intervals within the *Nba2* autoimmune susceptibility locus. J Immunol. (2010) 184:775–86. 10.4049/jimmunol.0901322 20018631 PMC2841050

[115] Cheung Y-H Landolt-Marticorena C Lajoie G Wither JE. The Lupus phenotype in B6.NZBc1 congenic mice reflects interactions between multiple susceptibility loci and a suppressor locus. Genes Immun. (2011) 12:251–62. 10.1038/gene.2010.71 21307879

[116] Morel L Blenman KR Croker BP Wakeland EK. The major murine systemic lupus erythematosus susceptibility locus, Sle1, is a cluster of functionally related genes. Proc Natl Acad Sci USA. (2001) 98:1787–92. 10.1073/pnas.98.4.1787 11172029 PMC29335

[117] Wandstrat AE Nguyen C Limaye N Chan AY Subramanian S Tian XH. Association of extensive polymorphisms in the SLAM/CD2 gene cluster with murine lupus. Immunity. (2004) 21:769–80. 10.1016/j.immuni.2004.10.009 15589166

[118] Veillette A. Immune regulation by SLAM family receptors and SAP-related adaptors. Nat Rev Immunol. (2006) 6:56–66. 10.1038/nri1761 16493427

[119] Schwartzberg PL Mueller KL Qi H Cannons JL. SLAM receptors and SAP influence lymphocyte interactions, development and function. Nat Rev Immunol. (2009) 9:39–46. 10.1038/nri2456 19079134

[120] Sintes J Bastos R Engel P SLAM Family Receptors and Autoimmunity, Autoimmune Disorders - Pathogenetic Aspects. Mavragani C, editor. Rijeka: InTech (2011). 10.5772/20641

[121] Cannons JL Yu LJ Jankovic D Crotty S Horai R Kirby M. SAP regulates T cell-mediated help for humoral immunity by a mechanism distinct from cytokine regulation. J Exp Med. (2006) 203:1551–65. 10.1084/jem.20052097 16754717 PMC2118305

[122] Crotty S Kersh EN Cannons J Schwartzberg PL Ahmed R. SAP is required for generating long-term humoral immunity. Nature. (2003) 421:282–7. 10.1038/nature01318 12529646

[123] Ma CS Hare NJ Nichols KE Dupré L Andolfi G Roncarolo MG. Impaired humoral immunity in X-linked lymphoproliferative disease associated with defective IL-10 production by CD4+ T cells. J Clin Invest. (2005) 115:1049–59. 10.1172/JCI200523139 15761493 PMC1059448

[124] Graham DB Bell MP McCausland MM Huntoon CJ van Deursen J Faubion WA Ly9 (CD229)-deficient mice exhibit T cell defects yet do not share several phenotypic characteristics associated with SLAM- and SAP-deficient mice. J Immunol. (2006) 176:291–300. 10.4049/jimmunol.176.1.291 16365421

[125] Romero X Zapater N Calvo M Kalko SG de la Fuente MA Tovar V. CD229 (Ly9) lymphocyte cell surface receptor interacts homophilically through its N-terminal domain and relocalizes to the immunological synapse. J Immunol. (2005) 174:7033–42. 10.4049/jimmunol.174.11.7033 15905546

[126] Cannons JL Qi H Lu KT Dutta M Gomez-Rodriguez J Cheng J. Optimal germinal center responses require a multistage T cell:B cell adhesion process involving integrins, SLAM-associated protein, and CD84. Immunity. (2010) 32:253–65. 10.1016/j.immuni.2010.01.010 20153220 PMC2830297

[127] Qi H Cannons JL Klauschen F Schwartzberg PL Germain RN. SAP-controlled T-B cell interactions underlie germinal centre formation. Nature. (2008) 455:764–9. 10.1038/nature07345 18843362 PMC2652134

[128] Kumar KR Li L Yan M Bhaskarabhatla M Mobley AB Nguyen C. Regulation of B cell tolerance by the lupus susceptibility gene Ly108. Science. (2006) 312:1665–9. 10.1126/science.1125893 16778059

[129] Wong EB Soni C Chan AY Domeier PP Shwetank Abraham T. B cell-intrinsic CD84 and Ly108 maintain germinal center B cell tolerance. J Immunol. (2015) 194:4130–43. 10.4049/jimmunol.1403023 25801429 PMC4402266

[130] Morel L Tian XH Croker BP Wakeland EK. Epistatic modifiers of autoimmunity in a murine model of lupus nephritis. Immunity. (1999) 11:131–9. 10.1016/S1074-7613(00)80088-6 10485648

[131] Niederer HA Willcocks LC Rayner TF Yang W Lau YL Williams TN. Copy number, linkage disequilibrium and disease association in the FCGR locus. Hum Mol Genet. (2010) 19:3282–94. 10.1093/hmg/ddq216 20508037 PMC2908468

[132] Breunis WB van Mirre E Bruin M Geissler J de Boer M Peters M. Copy number variation of the activating FCGR2C gene predisposes to idiopathic thrombocytopenic purpura. Blood. (2008). 111:1029–38. 10.1182/blood-2007-03-079913 17827395

[133] Willcocks LC Lyons PA Clatworthy MR Robinson JI Yang W Newland SA. Copy number of FCGR3B, which is associated with systemic lupus erythematosus, correlates with protein expression and immune complex uptake. J Exp Med. (2008) 205:1573–82. 10.1084/jem.20072413 18559452 PMC2442635

[134] Zhou XJ Lv JC Bu DF Yu L Yang YR Zhao J. Copy number variation of FCGR3A rather than FCGR3B and FCGR2B is associated with susceptibility to anti-GBM disease. Int Immunol. (2010) 22:45–51. 10.1093/intimm/dxp113 19946017

[135] Smith KG Clatworthy MR. FcγRIIB in autoimmunity and infection: evolutionary and therapeutic implications. Nat Rev Immunol. (2010) 10:328–43. 10.1038/nri2762 20414206 PMC4148599

[136] Su K Wu J Edberg JC Li X Ferguson P Cooper GS. A promoter haplotype of the immunoreceptor tyrosine-based inhibitory motif-bearing FcγRIIb alters receptor expression and associates with autoimmunity. I. Regulatory FCGR2B polymorphisms and their association with systemic lupus erythematosus. J Immunol. (2004) 172:7186–91. 10.4049/jimmunol.172.11.7186 15153543

[137] Su K Li X Edberg JC Wu J Ferguson P Kimberly RP A promoter haplotype of the immunoreceptor tyrosine-based inhibitory motif-bearing FcγRIIb alters receptor expression and associates with autoimmunity. II. Differential binding of GATA4 and Yin-Yang transcription factors and correlated receptor expression and function. J Immunol. (2004) 172:7192–9. 10.4049/jimmunol.172.11.7192 15153544

[138] Su K Yang H Li X Gibson AW Cafardi JM Zhou T. Expression profile of FcγRIIb on leukocytes and its dysregulation in systemic lupus erythematosus. J Immunol. (2007) 178:3272–80. 10.4049/jimmunol.178.5.3272 17312177 PMC2824439

[139] Blank MC Stefanescu RN Masuda E Marti F King PD Redecha PB. Decreased transcription of the human FCGR2B gene mediated by the−343 G/C promoter polymorphism and association with systemic lupus erythematosus. Hum Genet. (2005) 117:220–7. 10.1007/s00439-005-1302-3 15895258

[140] Kyogoku C Dijstelbloem HM Tsuchiya N Hatta Y Kato H Yamaguchi A. Fcγ receptor gene polymorphisms in Japanese patients with systemic lupus erythematosus: contribution of FCGR2B to genetic susceptibility. Arthritis Rheum. (2002) 46:1242–54. 10.1002/art.10257 12115230

[141] Kono H Kyogoku C Suzuki T Tsuchiya N Honda H Yamamoto K. FcγRIIB Ile232Thr transmembrane polymorphism associated with human systemic lupus erythematosus decreases affinity to lipid rafts and attenuates inhibitory effects on B cell receptor signaling. Hum Mol Genet. (2005) 14:2881–92. 10.1093/hmg/ddi320 16115811

[142] Floto RA Clatworthy MR Heilbronn KR Rosner DR MacAry PA Rankin A. Loss of function of a lupus-associated FcγRIIb polymorphism through exclusion from lipid rafts. Nat Med. (2005) 11:1056–8. 10.1038/nm1288 16170323

[143] Chen JY Wang CM Ma CC Luo SF Edberg JC Kimberly RP. Association of a transmembrane polymorphism of Fcγ receptor IIb (FCGR2B) with systemic lupus erythematosus in Taiwanese patients. Arthritis Rheum. (2006) 54:3908–17. 10.1002/art.22220 17133600

[144] Siriboonrit U Tsuchiya N Sirikong M Kyogoku C Bejrachandra S Suthipinittharm P. Association of Fcγ receptor IIb and IIIb polymorphisms with susceptibility to systemic lupus erythematosus in Thais. Tissue Antigens. (2003) 61:374–83. 10.1034/j.1399-0039.2003.00047.x 12753656

[145] Chu ZT Tsuchiya N Kyogoku C Ohashi J Qian YP Xu SB. Association of Fcγ receptor IIb polymorphism with susceptibility to systemic lupus erythematosus in Chinese: a common susceptibility gene in the Asian populations. Tissue Antigens. (2004) 63:21–7. 10.1111/j.1399-0039.2004.00142.x 14651519

[146] Li X Wu J Carter RH Edberg JC Su K Cooper GS. A novel polymorphism in the Fcγ receptor IIB (CD32B) transmembrane region alters receptor signaling. Arthritis Rheum. (2003) 48:3242–52. 10.1002/art.11313 14613290

[147] Willcocks LC Carr EJ Niederer HA Rayner TF Williams TN Yang W. A defunctioning polymorphism in FCGR2B is associated with protection against malaria but susceptibility to systemic lupus erythematosus. Proc Natl Acad Sci USA. (2010) 107:7881–5. 10.1073/pnas.0915133107 20385827 PMC2867866

[148] Lee YH Ji JD Song GG. Fcγ receptor IIB and IIIB polymorphisms and susceptibility to systemic lupus erythematosus and lupus nephritis: a meta-analysis. Lupus. (2009) 18:727–34. 10.1177/0961203309104020 19502269

[149] Zhu XW Wang Y Wei YH Zhao PP Wang XB Rong JJ. Comprehensive Assessment of the Association between FCGRs polymorphisms and the risk of systemic lupus erythematosus: evidence from a meta-analysis. Sci Rep. (2016) 6:31617. 10.1038/srep31617 27538381 PMC4990922

[150] Chen JY Wang CM Ma CC Hsu LA Ho HH Wu YJ. A transmembrane polymorphism in FcγRIIb (FCGR2B) is associated with the production of anti-cyclic citrullinated peptide autoantibodies in Taiwanese RA. Genes Immun. (2008) 9:680–8. 10.1038/gene.2008.56 18633424 PMC3740516

[151] Clatworthy MR Willcocks L Urban B Langhorne J Williams TN Peshu N. Systemic lupus erythematosus-associated defects in the inhibitory receptor FcγRIIb reduce susceptibility to malaria. ProcNatlAcadSci USA. (2007) 104:7169–74. 10.1073/pnas.0608889104 17435165 PMC1855357

[152] Morel L. Genetics of SLE: evidence from mouse models. Nat Rev Rheumatol. (2010) 6:348–57. 10.1038/nrrheum.2010.63 20440287

[153] Cunninghame Graham DS Vyse TJ Fortin PR Montpetit A Cai YC Lim S. Association of LY9 in UK and Canadian SLE families. Genes Immun. (2008) 9:93–102. 10.1038/sj.gene.6364453 18216865

[154] Suarez-Gestal M Calaza M Endreffy E Pullmann R Ordi-Ros J Sebastiani GD. Replication of recently identified systemic lupus erythematosus genetic associations: a case-control study. Arthritis Res Ther. (2009) 11:R69. 10.1186/ar2698 19442287 PMC2714115

[155] Suzuki A Yamada R Kochi Y Sawada T Okada Y Matsuda K. Functional SNPs in CD244 increase the risk of rheumatoid arthritis in a Japanese population. Nat Genet. (2008) 40:1224–9. 10.1038/ng.205 18794858

[156] Ota Y Kawaguchi Y Takagi K Tochimoto A Kawamoto M Katsumata Y. Single nucleotide polymorphisms of CD244 gene predispose to renal and neuropsychiatric manifestations with systemic lupus erythematosus. Mod Rheumatol. (2010) 20:427–31. 10.1007/s10165-010-0302-x 20437071

[157] Harley IT Kaufman KM Langefeld CD Harley JB Kelly JA. Genetic susceptibility to SLE: new insights from fine mapping and genome-wide association studies. Nat Rev Genet. (2009) 10:285–90. 10.1038/nrg2571 19337289 PMC2737697

[158] Theofilopoulos AN Kono DH Baccala R. The multiple pathways to autoimmunity. Nat Immunol. (2017) 18:716–24. 10.1038/ni.3731 28632714 PMC5791156

[159] Wandstrat AE Wakeland E. The genetics of complex autoimmune diseases: non-MHC susceptibility genes. Nat Immunol. (2001) 2:802–9. 10.1038/ni0901-802 11526390

[160] Mooney MA Nigg JT McWeeney SK Beth Wilmot B. Functional and genomic context in pathway analysis of GWAS data. Trends Genet. (2014) 30:390–400. 10.1016/j.tig.2014.07.004 25154796 PMC4266582

[161] Takai T Ono M Hikida M Ohmori H Ravetch JV. Augmented humoral and anaphylactic responses in Fcγ RII-deficient mice. Nature. (1996) 379:346–9. 10.1038/379346a0 8552190

[162] Nimmerjahn F Ravetch JV. Antibody-mediated modulation of immune responses. Immunol Rev. (2010) 236:265–75. 10.1111/j.1600-065X.2010.00910.x 20636822

[163] Soni C Domeier PP Wong EB Shwetank Khan TN Elias MJ. Distinct and synergistic roles of FcγRIIB deficiency and 129 strain-derived SLAM family proteins in the development of spontaneous germinal centers and autoimmunity. J Autoimmun. (2015) 63:31–46. 10.1016/j.jaut.2015.06.011 26162758 PMC4564365

[164] Yuasa T Kubo S Yoshino T Ujike A Matsumura K Ono M. Deletion of fcγ receptor IIB renders H-2(b) mice susceptible to collagen-induced arthritis. J Exp Med. (1999) 189:187–94. 10.1084/jem.189.1.187 9874575 PMC1887699

[165] Nakamura A Yuasa T Ujike A Ono M Nukiwa T Ravetch JV. Fcγ receptor IIB-deficient mice develop Goodpasture's syndrome upon immunization with type IV collagen: a novel murine model for autoimmune glomerular basement membrane disease. J Exp Med. (2000) 191:899–906. 10.1084/jem.191.5.899 10704470 PMC2195851

[166] van den Berghe T Hulpiau P Martens L Vandenbroucke RE Van Wonterghem E Perry SW Passenger mutations confound interpretation of all genetically modified congenic mice. Immunity. (2015) 43:200–9. 10.1016/j.immuni.2015.06.011 26163370 PMC4800811

[167] Shlomchik MJ Marshak-Rothstein A Wolfowicz CB Rothstein TL Weigert MG. The role of clonal selection and somatic mutation in autoimmunity. Nature. (1987) 328:805–11. 10.1038/328805a0 3498121

[168] Shlomchik M Mascelli M Shan H Radic MZ Pisetsky D Marshak-Rothstein A. Anti-DNA antibodies from autoimmune mice arise by clonal expansion and somatic mutation. J Exp Med. (1990) 171:265–92. 10.1084/jem.171.1.265 2104919 PMC2187662

[169] van Es JH Gmelig Meyling FH van de Akker WR Aanstoot H Derksen RH Logtenberg T. Somatic mutations in the variable regions of a human IgG anti-double-stranded DNA autoantibody suggest a role for antigen in the induction of systemic lupus erythematosus. J Exp Med. (1991) 173:461–70. 10.1084/jem.173.2.461 1899104 PMC2118793

[170] Winkler TH Fehr H Kalden JR. Analysis of immunoglobulin variable region genes from human IgG anti-DNA hybridomas. Eur J Immunol. (1992) 22:1719–28. 10.1002/eji.1830220709 1623920

[171] Wellmann U Letz M Herrmann M Angermuller S Kalden JR Winkler TH. The evolution of human anti-double-stranded DNA autoantibodies. Proc Natl Acad Sci USA. (2005) 102:9258–63. 10.1073/pnas.0500132102 15968001 PMC1166593

[172] Mietzner B Tsuiji M Scheid J Velinzon K Tiller T Abraham K. Autoreactive IgG memory antibodies in patients with systemic lupus erythematosus arise from nonreactive and polyreactive precursors. Proc Natl Acad Sci USA. (2008) 105:9727–32. 10.1073/pnas.0803644105 18621685 PMC2474524

[173] Tiller T Kofer J Kreschel C Busse CE Riebel S Wickert S Development of self-reactive germinal center B cells and plasma cells in autoimmune FcγRIIB-deficient mice. J Exp Med. (2010) 207:2767–78. 10.1084/jem.20100171 21078890 PMC2989760

[174] Li H Jiang Y Prak EL Radic M Weigert M. Editors and editing of anti-DNA receptors. Immunity. (2001) 15:947–57. 10.1016/S1074-7613(01)00251-5 11754816

[175] Radic MZ Weigert M. Genetic and structural evidence for antigen selection of anti-DNA antibodies. Annu Rev Immunol. (1994) 12:487–520. 10.1146/annurev.iy.12.040194.002415 8011289

[176] Sekiguchi DR Jainandunsing SM Fields ML Maldonado MA Madaio MP Erikson J. Chronic graft-versus-host in Ig knockin transgenic mice abrogates B cell tolerance in anti-double-stranded DNA B cells. J Immunol. (2002) 168:4142–53. 10.4049/jimmunol.168.8.4142 11937575

[177] Sekiguchi DR Eisenberg RA Weigert M. Secondary heavy chain rearrangement: a mechanism for generating anti-double-stranded DNA B cells. J Exp Med. (2003) 197:27–39. 10.1084/jem.20020737 12515811 PMC2193805

[178] Fukuyama H Nimmerjahn F Ravetch JV. The inhibitory Fcγ receptor modulates autoimmunity by limiting the accumulation of immunoglobulin G+ anti-DNA plasma cells. Nat Immunol. (2005) 6:99–106. 10.1038/ni1151 15592473

[179] Rahman ZS Alabyev B Manser T FcγRIIB regulates autoreactive primary antibody-forming cell, but not germinal center B cell, activity. J Immunol. (2007) 178:897–907. 10.4049/jimmunol.178.2.897 17202351

[180] Yajima K Nakamura A Sugahara A Takai T. FcγRIIB deficiency with Fas mutation is sufficient for the development of systemic autoimmune disease. Eur J Immunol. (2003) 33:1020–9. 10.1002/eji.200323794 12672068

[181] Weisenburger T von Neubeck B Schneider A Ebert N Schreyer D Acs A. Epistatic Interactions Between Mutations of Deoxyribonuclease 1-Like 3 and the Inhibitory Fc Gamma Receptor IIB Result in Very Early and Massive Autoantibodies Against Double-Stranded DNA. Front Immunol. (2018) 9:1551. 10.3389/fimmu.2018.01551 30026744 PMC6041390

[182] McGaha TL Sorrentino B Ravetch JV. Restoration of tolerance in lupus by targeted inhibitory receptor expression. Science. (2005) 307:590–3. 10.1126/science.1105160 15681388

[183] Brownlie RJ Lawlor KE Niederer HA Cutler AJ Xiang Z Clatworthy MR. Distinct cell-specific control of autoimmunity and infection by FcγRIIb. J Exp Med. (2008) 205:883–95. 10.1084/jem.20072565 18362174 PMC2292226

[184] Simon MM Greenaway S White JK Fuchs H Gailus-Durner V Wells S A comparative phenotypic and genomic analysis of C57BL/6J and C57BL/6N mouse strains. Genome Biol. (2013) 4:R82 10.1186/gb-2013-14-7-r82 PMC4053787 23902802

[185] Ravetch JV Nimmerjahn F Carroll MC Fc and complement receptors. In: Alt F Honjo T Radbruch A Reth M editors. Molecular Biology of B Cells. Cambridge: Academic Press (2015). p. 171–86. 10.1016/B978-0-12-397933-9.00011-4

[186] Sharp PE Martin-Ramirez J Boross P Mangsbo SM Reynolds J Moss J Increased incidence of anti-GBM disease in Fcγ receptor 2b deficient mice, but not mice with conditional deletion of Fcgr2b on either B cells or myeloid cells alone. Mol Immunol. (2012) 50:49–56. 10.1016/j.molimm.2011.12.007 22244885

[187] Yilmaz-Elis AS Ramirez JM Asmawidjaja P van der Kaa J Mus AM Brem MD. FcγRIIb on myeloid cells rather than on B cells protects from collagen-induced arthritis. J Immunol. (2014) 192:5540–7. 10.4049/jimmunol.1303272 24842758

[188] Lin Q Ohtsuji M Amano H Tsurui H Tada N Sato R. FcγRIIb on B cells and myeloid cells modulates B cell activation and autoantibody responses via different but synergistic pathways in lupus-prone Yaa mice. J Immunol. (2018) 201:3199–210. 10.4049/jimmunol.1701487 30373853

[189] Mildner A Giladi A David E Lara-Astiaso D Lorenzo-Vivas E Paul F. Genomic characterization of murine monocytes reveals C/EBPβ transcription factor dependence of Ly6C^−^ cells. Immunity. (2017) 46:849–62. 10.1016/j.immuni.2017.04.018 28514690

[190] Hirose S Lin Q Ohtsuji M Nishimura H Verbeek JS Monocyte subsets involved in the development of systemic lupus erythematosus and rheumatoid arthritis. Int Immunol. (2019) 31:dxz036 10.1093/intimm/dxz036 PMC6794944 31063541

[191] Biesen R Demir C Barkhudarova F Grün JR Steinbrich-Zollner M Backhaus M. Sialic acid-binding Ig-like lectin 1 expression in inflammatory and resident monocytes is a potential biomarker for monitoring disease activity and success of therapy in systemic lupus erythematosus. Arthritis Rheum. (2008) 58:1136–45. 10.1002/art.23404 18383365

[192] Cros J Cagnard N Woollard K Patey N Zhang SY Senechal B. Human CD14dim monocytes patrol and sense nucleic acids and viruses via TLR7 and TLR8 receptors. Immunity. (2010) 33:375–86. 10.1016/j.immuni.2010.08.012 20832340 PMC3063338

